# A hybrid adaptive large neighborhood search for time-dependent open electric vehicle routing problem with hybrid energy replenishment strategies

**DOI:** 10.1371/journal.pone.0291473

**Published:** 2023-09-14

**Authors:** Lijun Fan

**Affiliations:** School of Management, Hunan University of Technology and Business, Changsha, China; University of Victoria / Universiti Teknologi Malaysia /, CANADA

## Abstract

As competition intensifies, an increasing number of companies opt to outsource their package distribution operations to professional Third-Party Logistics (3PL) fleets. In response to the growing concern over urban pollution, 3PL fleets have begun to deploy Electric Vehicles (EVs) to perform transportation tasks. This paper aims to address the Time-Dependent Open Electric Vehicle Routing Problem with Hybrid Energy Replenishment Strategies (TDOEVRP-HERS) in the context of urban distribution. The study considers the effect of dynamic urban transport networks on EV energy drain and develops an approach for estimating energy consumption. Meanwhile, the research further empowers 3PL fleets to judiciously oscillate between an array of energy replenishment techniques, encompassing both charging and battery swapping. Based on these insights, a Mixed-Integer Programming (MIP) model with the objective of minimizing total distribution costs incurred by the 3PL fleet is formulated. Given the characteristics of the model, a Hybrid Adaptive Large Neighborhood Search (HALNS) is designed, synergistically integrating the explorative prowess of Ant Colony Optimization (ACO) with the localized search potency of Adaptive Large Neighborhood Search (ALNS). The strategic blend leverages the broad-based solution initiation of ACO as a foundational layer for ALNS’s deeper, nuanced refinements. Numerical experiments on a spectrum of test sets corroborate the efficacy of the HALNS: it proficiently designs vehicular itineraries, trims down EV energy requisites, astutely chooses appropriate energy replenishment avenues, and slashes logistics-related outlays. Therefore, this work not only introduces a new hybrid heuristic technique within the EVRP field, providing high-quality solutions but also accentuates its pivotal role in fostering a sustainable trajectory for urban logistics transportation.

## 1. Introduction

Amid the continuous development of the economy and the accelerated pace of urbanization, the demand for urban logistics distribution is growing rapidly [1]. Traditional urban logistics predominantly relies on Fuel Vehicles (FV), leading to high fuel consumption, severe noise pollution, and significant carbon emissions, which exacerbate urban environmental degradation [2]. In contrast, Electric Vehicles (EVs), characterized by low energy consumption, reduced noise, and minimal pollution, have emerged as an optimal solution for urban distribution and are increasingly recognized as essential tools in transport activities [3]. As per International Energy Agency [4], global adoption rates of EVs have tripled in the past three years. Projections from McKinsey & Company [5] suggest that by Year 2030, 10 to 50 percent of new vehicle sales in urban settings will be electric. In alignment with this trend, leading global logistics companies are actively promoting electrified transportation fleets. For instance, FedEx aims to achieve over 50% of its distribution volume using EVs worldwide by 2030 and plans to replace all transport vehicles with electric alternatives by 2040 [6]. Similarly, DHL intends to operate 14,000 EVs globally by 2025, representing approximately one-third of its entire fleet [7]. These data emphasize the salience of the Electric Vehicle Routing Problem (EVRP) research in shaping urban logistics in response to evolving societal dynamics.

However, EVs face significant limitations in urban distribution compared to traditional FVs. The prominent factors include a limited travel range [8] and long charging durations [9]. Urban traffic networks are complex and changeable, with congestion occurring from time to time, leading to varying vehicle speeds that impact the power consumption by the battery and, thus, the vehicle’s mileage. Consequently, estimating the remaining real-time range during EV journeys poses a substantial challenge to distribution route planning [10]. While charging en route can mitigate range anxiety, the current charging technology extends EV charging time greatly beyond those required for refueling FVs, significantly impacting delivery efficiency. Hence, the battery swapping strategy becomes an effective solution to offset the lengthy charging time. By establishing dedicated battery swapping stations, logistics fleets can replace EV batteries within 10 minutes [11], markedly reducing energy replenishment durations. With the gradual improvement of urban charging infrastructure, transportation fleets have begun to adopt different energy replenishment methods according to actual situations. They opt for charging stations when the recharging demand is low to save on distribution costs, while selecting battery swapping stations when the recharging demand is high to enhance delivery efficiency. These different strategies have varying implications for EV distribution costs and efficiency [12]. In summary, when optimizing EV urban logistics distribution routes, planners must consider the time-varying factors of the urban traffic network and choose appropriate energy supplement methods for EVs based on different scenarios.

In addition, in today’s fiercely competitive market milieu, companies must strive to reduce operating costs to gain a competitive edge. One effective strategy to achieve this target is to hire professional Third-Party Logistics (3PL) fleets for urban distribution [13]. The Open Vehicle Routing Problem (OVRP) exemplifies this cooperative mode. A unique feature of OVRP is that after the completion of delivery tasks, the 3PL fleets do not need to return to the enterprise’s depot, which greatly reduces unnecessary empty-load travel and effectively reduces distribution costs [14]. When devising a solution for open vehicle distribution, replacing traditional FVs with EVs not only saves energy costs but also reduces carbon emissions, aligning with the expectations of social development. Currently, there are few studies on the Open Electric Vehicle Routing Problem (OEVRP), making it highly significant for research.

Based on the aforementioned description, this research integrates authentic factors, including the dynamic nature of urban traffic networks, diverse energy replenishment strategies, and 3PL fleets delivery, into the Electric Vehicle Routing Problem (EVRP). In this context, a novel Time-Dependent Open Electric Vehicle Routing Problem with Hybrid Energy Replenishment Strategies (TDOEVRP-HERS) is proposed to minimize the distribution cost of 3PL fleets paid by the enterprise. Unlike previous research, this study considers elements such as EV self-weight, real-time package load, travel speed, and travel distance when predicting EV energy consumption. It develops methods for estimating EV power consumption and travel time in the dynamic transportation network. To solve the TDOEVRP-HERS, a hybrid metaheuristic combining Ant Colony Optimization (ACO) and Adaptive Large Neighborhood Search (ALNS) is designed.

The main contributions of this study are summarized as follows: (1) TDOEVRP-HERS is studied in this paper, filling a research gap. (2) A method for estimating EV energy consumption in time-varying traffic networks is devised, aiding logistics fleets to better perform EV urban distribution. (3) The study develops a flexible hybrid energy replenishment strategy that improves delivery efficiency and reduces the distribution cost of logistics fleets by selecting the charging or battery swapping option based on different circumstances. (4) The research pioneers the combined use of ACO and ALNS specifically in the context of EVRPs. Despite each algorithm’s individual effectiveness in solving various optimization problems, the exploration of their combined application has remained relatively uncharted. This study leverages the potential synergy between these two algorithms, representing an innovative contribution to the existing body of research. The results demonstrate that the Hybrid Adaptive Large Neighborhood Search (HALNS) offers enhanced problem-solving capabilities, thereby establishing a promising new direction in the field of TDOEVRP. (5) The paper evaluates the performance of the proposed algorithm through extensive numerical experiments and examines the economic and environmental advantages of TDOEVRP-HERS. The experimental findings offer valuable insights and recommendations to governmental policymakers and logistics fleet managers from multiple perspectives, facilitating efforts to promote energy conservation and emission reduction within the logistics sector.

The remainder of this article unfolds as follows: The Section 2 encompasses a review of pertinent literature and elaborates on the innovative aspects of this study. The Section 3 introduces the theoretical methodology for estimating EV power consumption and travel time in the dynamic urban traffic network. The Section 4 formally defines the research question and develops a Mixed-Integer Programming (MIP) model for the problem. The Section 5 details the design of the HALNS. In the Section 6, a sequence of numerical experiments is conducted to validate the efficacy and rationality of the proposed algorithm, accompanied by practical recommendations related to sustainable urban distribution. Finally, the Section 7 encapsulates the conclusion of this study.

## 2. Related literature review

As a well-known Combinatorial Optimization Problem (COP), the Vehicle Routing Problem (VRP) aims to determine the optimal set of routes for delivering packages from the depot to customers based on various objective functions [15]. Currently, researchers have applied VRPs to distinct transportation scenarios, such as urban waste recycling [16], post-earthquake relief distribution [17], and vaccine emergency response [18]. This paper delves into urban distribution through the lens of 3PL fleets. The introduced TDOEVRP-HERS, a VRP variant, integrates time-varying vehicle speeds, varied energy replenishment strategies, and open routing. This section offers a literature review concerning these elements and the metaheuristics employed to address such challenges.

### 2.1. Research on the Time-Dependent Vehicle Routing Problem (TDVRP)

The urban transportation network exhibits time-varying dynamic nature, wherein vehicular velocities may fluctuate across varying time periods. This variation significantly affects the EV energy consumption and travel time cost of urban logistics distribution. Scholars initially focused on TDVRP, which involved studying the real-time speed impact of different weather and congestion levels on vehicle routing [19], identifying ways to evade traffic congestion by analyzing urban traffic’s time-varying characteristics [20], and addressing the transport of valuable goods by proposing a secure TDVRP with time windows including pickup and delivery with uncertain demands [21]. Researchers then discovered that time-varying vehicle speeds caused various carbon emissions due to different fuel consumption, leading to the development of the Time-Dependent Green Vehicle Routing Problem (TDGVRP). Soysal et al. [22] and Çimen et al. [23] established the TDGVRP model, which considered time-varying vehicle speeds, fuel consumption, and carbon emissions. TDGVRP was later focused on cold chain transportation by Guo et al. [24], who proposed a two-stage algorithm that enabled vehicles to wait in place after service to avoid harsh traffic conditions. Recently, with the popularity of EV fleets, the Time-Dependent Electric Vehicle Routing Problem (TDEVRP) is rising gradually. TDEVRP is, in fact, a subset of TDGVRP, which considers time-varying vehicle speeds’ impact on EVs’ energy consumption rather than FVs’ fuel consumption. Lu et al. [25] considered the impact of charging decisions and congestion conditions on the overall delivery process based on traditional constraints in TDEVRP. Bi and Tang [26] examined TDEVRP by incorporating time-varying random traffic conditions and employing the analytical battery model to determine EVs’ charging and discharging patterns. They aimed to minimize the overall service time and developed a hybrid rollout algorithm to tackle the problem. Zhang et al. [10] and Keskin et al. [27] approached TDEVRP from different viewpoints. The former incorporated charging during congestion periods into the objective function, while the latter considered the impact of queuing time at charging stations on overall distribution. The literature above provides a theoretical framework and solution methodology for TDEVRP.

### 2.2. Research on the Open Vehicle Routing Problem (OVRP)

OVRP has found practical application in a variety of scenarios, such as newspaper distribution [28], express service [29], campus shuttle [30], and home health care [31]. Scholars have examined the OVRP from diverse perspectives considering different constraints. Atefi et al. [32] developed a unique OEVRP model that utilized multiple carriers to deliver packages for a Canadian major producer of cookies and snacks. Azadeh et al. [33] introduced a closed-open hybrid VRP based on the cooperation model of self-operated fleets and 3PL fleets, allowing some vehicles to return to the depot, while the rest of the vehicles do not. Niu et al. [34] discussed the OVRP of outsourcing distribution to a 3PL fleet, and demonstrated the advantages of outsourcing logistics through cost composition analysis. Brandão [35] studied a multi-depot OVRP with the goal of minimizing the travel distance of vehicles. Xia and Fu [36] introduced customer satisfaction rate into the OVRP as a constraint, and constructed an optimization model to minimize the total distribution costs. In addition, many scholars have designed various algorithms for OVRP. Faiz et al. [37] presented two integer linear programming models for OVRP. The first one is an arc-based mixed integer linear programming model solved by a general-purpose solver, while the second one is based on a path-based formulation, which solved by a column generation framework. Chen et al. [38] developed an urgency level-based insertion heuristic to construct an initial solution, and employed a reinforcement learning based variable neighborhood search algorithm to solve the OVRP. Brandão [39] proposed an iterated local search algorithm for OVRP, and solved it with test sets including Solomon, Homberger and Gehring. With the rise of green logistics in recent years, the Green Open Vehicle Routing Problem (GOVRP) has gradually attracted attention. Niu et al. [40] considered vehicle fuel consumption and its environmental impact in GOVRP, conducted a simulation experiment based on the actual road conditions in Beijing, and found that compared to closed routes, open routes reduced total cost by 20%, with fuel consumption costs and carbon emission costs lowered by almost 30%. The above literature provides methodological guidance for OEVRP. However, there is a dearth of literature on OEVRP, indicating that related research has not received sufficient attention.

### 2.3. Research on energy replenishment strategies in EVRP

Efficient energy supplement strategies have always been a prominent research topic in EVRP, aiming to alleviate range anxiety and enhance delivery efficiency in EV distribution. Initially, scholars mainly focused on charging strategies, such as full charging, which has been utilized in the studies of Lin et al. [41], Granada-Echeverri et al. [42], and Kucukoglu et al. [43]. Full charging strategy involves completely charging the EV before departing from the charging station, which can effectively mitigate range anxiety. However, this strategy incurs high charging costs and long charging durations, may easily leading to a decrease in customer satisfaction. The strategy of partial charging, in its assessment of charging costs and service timeliness, affords greater flexibility in charging operations. Cortés-Murcia et al. [44] and Zhou et al. [45] presented partial charging strategies for EVs when designing EVRP charging schemes. The antecedent enables EVs to be dispatched for multiple distribution tasks, whereas the latter deftly employs the partial charging period to facilitate the delivery of parcels to clients closer to the charging station. In addition, there are different charging technologies in the charging process. Schiffer and Walther [46] and Dönmez et al. [47] proposed a linear charging function when designing the EVRP charging scheme. The former considers a single fast-charging mode at the charging station, while the latter considers a combination of three charging speeds: ordinary charging, fast charging, and super-fast charging. In recent years, battery swapping has emerged as a viable solution for energy replenishment in EV distribution. Jie et al. [48] and Zhou et al. [49] discussed the EVRP considering battery swapping strategy. The former developed a hybrid algorithm to tackle the model, while the latter proposed a multi-objective whale optimization algorithm for the model. Moreover, Raeesi and Zografos [50] devised a synchronized mobile battery swapping strategy for the EVRP, involving the replacement of depleted batteries with fully charged ones at designated times and locations by battery-swapping vans. The literature above can offer theoretical references and methodological support for EVs’ energy replenishment strategy in distribution.

### 2.4. Research on metaheuristics of solving VRP

The application of efficient meta-heuristic algorithms to optimize routing problems has been a focus of research in logistics transportation [51]. The TDOEVRP-HERS poses unique challenges due to the time-dependent nature of its constraints, alongside the specifics of 3PL fleets, such as non-mandatory return trips, flexible energy replenishment options, and uncertain battery level consumption. Two algorithmic strategies that have shown promise in addressing these complexities are the ACO [52] and ALNS [53]. ACO is a population-based metaheuristic that draws inspiration from the foraging behavior of ants [54]. Its utilization in complex VRPs has received extensive research attention. Su and Fan [55] used ACO to minimize penalty costs in the Green Vehicle Routing Problem (GVRP), incorporating fuel consumption, carbon emissions, and customer satisfaction constraints. Similarly, Zhang et al. [56] utilized ACO to cut down energy usage in EVRP. Despite its successes, the original ACO has shortcomings, leading researchers to develop the improved ACO. Zhang et al. [57] enhanced traditional ACO with three mutation operators for the multi-objective VRP with flexible time windows, improving local search and global exploration. Li et al. [58] adjusted pheromone update methods of ACO, focusing on optimal solutions for the multi-objective multi-depot GVRP. Jia et al. [59] created a bi-level ACO for EVRP with capacity constraints. The upper level, unconstrained by electricity, generates routes via ACO, while the lower level uses a heuristic to determine charging plans, using its solutions to update the upper level’s pheromone. ALNS is a well-regarded metaheuristic that has successfully addressed numerous COPs. It operates by iteratively exploring a set of candidate solutions in the neighborhood of the current solution and adaptively selecting the most promising ones [60]. Scholars have employed ALNS to solve many VRP variants. Sun et al. [61] addressed the time-dependent profitable pickup and delivery problem with time windows by ALNS. Chen et al. [62] discussed a VRP with time windows and delivery robots and developed ALNS to tackle it. Kuhn et al. [63] used the ALNS to integrate order picking and vehicle routing for micro-store supply. ACO is known for generating high-quality initial solutions that respect the problem’s constraints. ALNS is particularly effective when dealing with dynamic environments, given its capacity to continually adjust and refine solutions [64]. The combination of ACO’s global exploration and ALNS’s local exploitation may lead to better-quality solutions. The comprehensive search process increases the chance of finding optimal or near-optimal solutions for the TDOEVRP-HERS. However, the hybrid application of these two powerful algorithms in the field of EVRP is currently under-researched, suggesting a need for further exploration.

### 2.5. The research gaps

Table 1 presents a structured categorization of the literature pertinent to TDOEVRP-HERS, drawing from the elements outlined previously. This table systematically delineates distinctions among energy replenishment strategies, routing modes, and solution methodologies within existing studies. Its design facilitates an efficient understanding of the prevailing state-of-the-art and highlights potential research gaps or avenues addressed by this research.

**Table 1:** A summary of relevant works.

Year	Authors	Constraints							Solution method			
		EV	FV	TDVS	CR	OR	CS	BSS	ACbA	NbA	HM	others
2016	Lin et al. [41]	√			√		√					√
2017	Schiffer and Walther [46]	√			√		√					√
2018	Bi and Tang [26]	√		√	√		√					√
2018	Atefi et al. [32]		√			√				√		
2018	Niu et al. [34]		√			√				√	√	
2018	Zhang et al. [56]	√			√		√		√			
2019	Keskin et al. [27]	√		√	√		√			√		
2019	Azadeh et al. [33]		√		√	√					√	
2019	Xia and Fu [36]		√			√						√
2019	Faiz et al. [37]		√			√						√
2019	Jie et al. [48]	√			√			√		√	√	
2019	Zhang et al. [57]		√		√				√		√	
2020	Lu et al. [25]	√		√	√		√			√		
2020	Zhang et al. [10]	√		√	√		√			√		
2020	Brandão [35]		√			√				√		
2020	Chen et al. [38]		√			√				√		
2020	Su and Fan [55]		√		√				√			
2020	Sun et al. [61]		√	√	√					√		
2021	Kucukoglu et al. [43]	√			√		√					√
2021	Zhou et al. [45]	√			√		√			√	√	
2021	Chen et al. [62]		√		√					√		
2021	Kuhn et al. [63]		√		√					√		
2022	Guo et al. [24]		√	√	√					√	√	
2022	Dönmez et al. [47]	√	√		√		√			√		
2022	Zhou et al. [49]	√			√			√				√
2022	Jia et al. [59]	√			√		√		√			
-	This work	√		√		√	√	√	√	√	√	

* EV: Electric Vehicle. FV: Fuel Vehicle. TDVS: Time-Dependent Vehicle Speed. CR: Closed Routing. OR: Open Routing. CS: Charging Strategy. BSS: Battery Swapping Strategy. ACbA: Ant Colony-based Algorithm. NbA: Neighborhood-based Algorithm. HM: Hybrid Metaheuristics.

The existing literature lays a solid groundwork for the in-depth investigation of EVRP in urban distribution. However, a review of the literature has identified several gaps in research that merit further exploration: (1) Many studies limit EVs to using charging stations as their sole energy replenishment way during distribution. However, in reality, EV fleets have started to adopt a variety of energy replenishment strategies in current urban distribution. As one of the world’s largest consumers of EVs, China explicitly outlined its intention in the “New Energy Vehicle Industry Development Plan (2021–2035)” to “accelerate the construction of battery swap infrastructures,” indicating that battery swapping will become a primary method of supplementing EV energy in the future. Despite some scholars have explored EVRP with battery swapping, there remains a significant shortage of EVRP research that considers hybrid energy replenishment strategies with both charging and battery swapping. (2) The time-varying traffic conditions of urban road networks lead to EVs traveling at varying speeds throughout the day, with speed being the most significant factor affecting both EV travel time and power consumption. The fluctuating vehicle speed makes calculating EV energy consumption and remaining mileage challenging, thereby complicating the planning of urban distribution routes. Currently, the academic community has not given sufficient attention to developing reasonable rules for predicting EV energy consumption during urban distribution. (3) To reduce operational costs, an increasing number of enterprises are outsourcing their logistics transportation business. Logistics outsourcing involves a 3PL fleet that departs from the enterprise’s depot to perform distribution tasks without having to return to the depot upon completing the tasks, known as open route distribution. Although this cooperation mode is gaining popularity in urban distribution, there is a dearth of literature on TDOEVRP, which warrants further exploration. To fill these research gaps, this study investigates TDOEVRP-HERS, considering customer demand, service time, EV capacity, time-varying speed, different energy supplement strategies, and open routing. A MIP model is constructed to minimize the total distribution cost of transport fleets, and a HALNS is designed to tackle this model. The study’s results provide decision-making references for companies interested in hiring environmental-friendly 3PL fleets and governments seeking to promote sustainable urban logistics.

## 3. Calculation of EV power consumption and travel time in a dynamic transportation network

The road network in urban areas is characterized by its time-varying nature, meaning that the speed of vehicles may fluctuate during various time periods when traveling on urban roads. The time taken to traverse the road segment (*i*,*j*) depends not only on the road distance but also on the EV’s starting time, traveling speed, and real-time load. The vehicle may require several time periods to travel from node *i* to node *j*. Therefore, this study refers to the method proposed by Liu et al. [20] to partition a day of 24 hours into multiple equal time periods. *H* represents the length of a time period, and *n* represents the number of time periods in a day, i.e., *n* = 24/*H*. *T* represents the set of time periods in a day, i.e., $T=\{[0,T_{1}],[T_{1},T_{2}],...,[T_{n-1},T_{n}]\}$, where [0,*T*_1_] represents the first time period of a day, and [*T*_*n*−1_,*T*_*n*_] represents the *n*-th time period. Additionally, [*T*_*R*−1_,*T*_*R*_] signifies the *R*-th time period of a day, where *R*∈{1,2,…,*n*−1,*n*}, and *T*_*R*_−*T*_*R*−1_ = *H*. The traveling speed of EV *k* on road segment (*i*,*j*) during the *R*-th time period is denoted as $v_{ijk}^{R}$. By combining the EV energy consumption calculation methods proposed by Goeke and Schneider [65] and Basso et al. [8], the energy consumption $e_{ijk}^{R}$ generated by EV *k* when traveling at speed $v_{ijk}^{R}$ on road segment (*i*,*j*) during the *R*-th time period can be calculated as follows:

$$e_{ijk}^{R}=\phi^{d}\varphi^{d}(\frac{\left[g\sin\theta_{ij}+C_{r}\cdot g\cos\theta_{ij}](L+u_{ijk}\right)}{3600}+\frac{R_{c}A_{w}\rho {\left(v_{ijk}^{R}\right)}^{2}}{76140})v_{ijk}^{R}t_{ijk}^{R}$$ (1)

where *ϕ*^*d*^ denotes the output efficiency parameter of the driving motor, *φ*^*d*^ denotes the output efficiency parameter of the battery. Other factors influencing EV energy consumption include the rolling resistance coefficient (*C*_*r*_), air resistance coefficient (*R*_*c*_), air density (*ρ*), and the windward area of the vehicle (*A*_*w*_). *θ*_*ij*_ is the slope of road segment (*i*,*j*), and *g* is the gravitational acceleration. *L* denotes the self-weight of the EV, and *u*_*ijk*_ denotes the remaining load of EV *k* traveling on road (*i*,*j*). $t_{ijk}^{R}$ is the travel time of EV *k* on road section (*i*,*j*) during the *R*-th time period.

Let *d*_*ij*_ denote the distance between node *i* and node *j*. Let $d_{ijk}^{R}$ denote the remaining distance that EV *k* still needs to travel on road segment (*i*,*j*) at the end of the *R*-th time period. Under the time-varying urban road network, the following are procedures to calculate the power consumption *E*_*ijk*_ and travel time *T*_*ijk*_ that EV *k* requires to traverse road segment (*i*,*j*):

**Procedure 1:** The power consumption and travel time generated by EV *k* during the *R*-th time period when it leaves node *i*. Let $\partial_{ik}^{R}$ denote the moment when EV *k* departs from node *i* within the *R*-th time period, i.e., $T_{R-1}\leq \partial_{ik}^{R}\leq T_{R}$. Therefore, the feasible travel time of EV *k* during the *R*-th time period is $T_{R}-\partial_{ik}^{R}$. Consequently, the distance traveled by EV *k* on road segment (*i*,*j*) during the *R*-th time period is $F_{ijk}^{R}=v_{ijk}^{R}(T_{R}-\partial_{ik}^{R})$. If $F_{ijk}^{R}\geq d_{ij}$, it implies that EV *k* has covered the distance between node *i* and node *j* within the *R*-th time period, hence $d_{ijk}^{R}=0$, $T_{ijk}=t_{ijk}^{R}=d_{ij}/v_{ijk}^{R}$, and $E_{ijk}=e_{ijk}^{R}$. The calculation process is complete. If $F_{ijk}^{R}<d_{ij}$, it signifies that EV *k* cannot travel from node *i* to node *j* during the *R*-th time period. Consequently, $d_{ijk}^{R}=d_{ij}-F_{ijk}^{R}$, and $t_{ijk}^{R}=T_{R}-\partial_{ik}^{R}$. Therefore, the energy consumption of EV *k* during the *R*-th time period is $e_{ijk}^{R}$. Proceed to Procedure 2.

**Procedure 2:** Computation of EV *k*‘s power consumption and travel time on road segment (*i*,*j*) after the *R*-th time period. Step 1: Set *ξ* = 1. Step 2: Calculate the possible travel distance $F_{ijk}^{R+\xi }$ of EV *k* during the (*R*+*ξ*)th time period, where $F_{ijk}^{R+\xi }=v_{ijk}^{R+\xi }\cdot H$. If $F_{ijk}^{R+\xi }<d_{ijk}^{R+\xi -1}$, it means that EV *k* still cannot travel to node *j* in the (*R*+*ξ*)th time period. Consequently, the travel time of EV *k* during the (*R*+*ξ*)th time period is $t_{ijk}^{R+\xi }=H$, and the remaining distance to node *j* is $d_{ijk}^{R+\xi }=d_{ijk}^{R+\xi -1}-F_{ijk}^{R+\xi }$. The energy consumption of EV *k* during this time period is $e_{ijk}^{R+\xi }$. Let *ξ* = *ξ*+1 and proceed to Step 2. If $F_{ijk}^{R+\xi }\geq d_{ijk}^{R+\xi -1}$, it means that EV *k* can travel to node *j* during the (*R*+*ξ*)th time period, then the travel time of EV *k* during this time period is $t_{ijk}^{R+\xi }=d_{ijk}^{R+\xi -1}/v_{ijk}^{R+\xi }$, and the energy consumption of EV *k* during this time period is $e_{ijk}^{R+\xi }$. Proceed to Procedure 3.

**Procedure 3:** Computation of the total power consumption and travel time for EV *k* traversing road segment (*i*,*j*). $E_{ijk}=\sum_{x=R}^{R+\xi }e_{ijk}^{x}$, $T_{ijk}=\sum_{x=R}^{R+\xi }t_{ijk}^{x}$. The calculation process is complete.

## 4. Problem and mathematical model

In this section, the TDOEVRP-HERS is formally defined, followed by a presentation of the mathematical formulation that captures the key objectives and constraints of the problem. Factors including energy replenishment strategies, time-dependent vehicle speed, open routing, real-time load, fixed EV usage costs, vehicle travel costs, customer service costs, and energy costs are considered. The goal is to minimize the aggregate of these costs by efficiently dispatching EVs from 3PL fleets and devising optimal vehicle routes.

### 4.1. Problem framework

This subsection introduces and analyzes the TDOEVRP-HERS in urban distribution. A retail company, lacking its own self-operated transportation fleets, delegates all package distribution tasks to a professional 3PL fleet. This 3PL fleet employs homogeneous EVs to deliver identical products to customers scattered all over a city. The objective is to minimize the total operating costs for the 3PL fleet, utilizing up to *K* vehicles. A complete graph *G* = {*V*,*A*} is defined where *V* = {0,1,…,*n*} is the set of all vertices, 0 stands for the depot, and *A* = {(*i*,*j*):*i*,*j*∈*V* and *i*≠*j*} is the set of arcs connecting nodes. Each arc (*i*,*j*)∈*A* has an associated distance *d*_*ij*_. Unlike other studies, the infrastructure for EV energy replenishment in this study is bifurcated into two types: Charging stations (*F*) and battery swapping stations (*B*). So, C=V\({0}∪F∪B) is the set of customer nodes.

**Fig 1:**
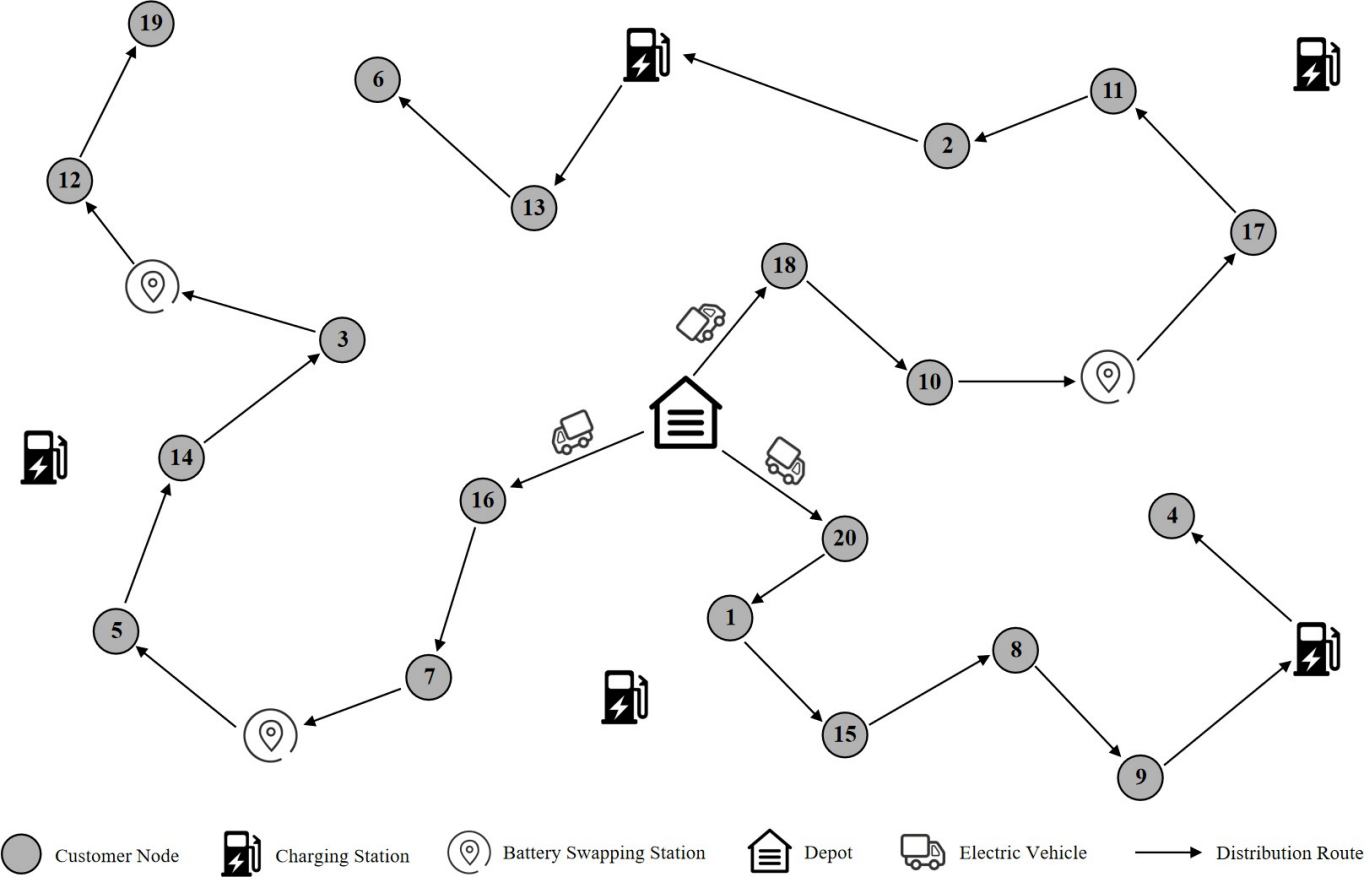
Schematic of the TDOEVRP-HERS.

Each customer *i*∈*C* is assigned one and only one EV to fulfill their delivery requirement, with the customer possessing a non-negative demand *D*_*i*_ and a designated service time *s*_*i*_. Specifically, before departing from the retail company’s depot, all EVs within the 3PL fleet load the maximum load capacity of *W* kilograms in goods for distribution. Concurrently, the EV fleet is fully charged and primed for action. During the transportation process, priority is accorded to EVs for customer service. However, should an EV find its energy insufficient to sustain the task, it must undertake an energy replenishment. According to the remaining delivery tasks, the EV can choose to replenish the battery level at either a charging station or a battery swapping station. Once the energy replenishment is completed, the EV resumes serving the remaining customers. Upon completion of all transportation tasks, the EV fleet can proceed to other enterprises for additional distribution tasks, without having to return to the retail company’s depot. The schematic of the TDOEVRP-HERS is depicted in Fig 1.

In this problem, all the necessary parameters, such as EVs’ maximal load *W* and battery capacity *Q*, depot coordinates, customers’ location, delivery volume, and service time, are known. The study aims to optimize the 3PL fleet’s transportation routes, while meeting all customers’ distribution demands, and minimizing the total distribution costs that the retail enterprise has to pay. Distribution costs include EV fixed dispatch costs, travel costs, customer service costs, as well as charging and battery swapping costs. Please see S1 Appendix for all the parameters and decision variables used.

### 4.2. Assumptions

To clearly construct the MIP model, the ensuing assumptions are defined: (1) Each EV in the 3PL fleet is dispatched at most once, and the delivery volume of each customer is less than EV capacity. (2) Urban traffic network affects EV speed, which varies with time. (3) The energy expenditure of EVs is affected by a gamut of factors encompassing vehicular self-weight, travel velocity, real-time load, and traversed distance. (4) Once an EV enters a charging station or battery swapping station, it can directly replenish energy without having to wait in line. To ensure the timeliness of distribution, the logistics fleet limits each EV to enter a charging station only once, whereas the number of times it enters a battery swapping station is unlimited. (5) The charging station adopts a full charging strategy with a constant charging rate, while the battery swapping station has a fixed battery swapping time and the battery level of EVs becomes full upon completion of battery swapping.

### 4.3. TDOEVRP-HERS model

Based on the problem description and variables mentioned earlier, the optimization model of TDOEVRP-HERS is built below:

$$\min\;P_{1}+P_{2}+P_{3}+P_{4}$$ (2)

$$P_{1}=c_{1}\sum_{i\in 0}\sum_{j\in C}\sum_{k\in K}x_{ijk}$$ (3)

$$P_{2}=c_{2}\sum_{i\in V}\sum_{j\in V}\sum_{k\in K}T_{ijk}x_{ijk}$$ (4)

$$P_{3}=c_{3}\sum_{i\in C}\sum_{k\in K}s_{i}w_{ik}$$ (5)

$$P_{4}=c_{4}\sum_{i\in F}\sum_{k\in K}\sigma_{ik}y_{ik}+c_{5}\sum_{j\in B}\sum_{k\in K}z_{jk}$$ (6)

The objective function (2) minimizes the total distribution cost for the 3PL fleet. This cost includes the fixed vehicle dispatching cost (*P*_1_), travel cost (*P*_2_), customer service cost (*P*_3_), and energy cost (*P*_4_). Notably, *P*_4_ is the sum of charging and battery swapping costs. Expressions for these costs within the framework of TDOEVRP-HERS are presented in Formulas (3) to (6).

In tandem with objective function (2), the study addresses various constraints, organized into five distinct groups: arc and vertex constraints, vehicle capacity constraints, battery level constraints, time constraints, and binary constraints. The arc and vertex constraints are presented through constraints (7) to (10), while vehicle capacity constraints are articulated by constraints (11) and (12). The battery level constraints are captured by constraints (13) to (18), and time-related restrictions are laid out in constraints (19) to (22). Lastly, constraint (23) enforces a binary constraint.


$$\sum_{i\in 0}\sum_{j\in C}x_{ijk}\leq 1,\forall k\in K$$ (7)

$$\sum_{k\in K}w_{ik}=1,\forall i\in C$$ (8)

$$\sum_{i\in V}x_{ijk}=\sum_{l\in V}x_{jlk},\forall j\in C\cup F\cup B,\forall k\in K,i\neq j,j\neq l$$ (9)

$$\sum_{i\in F}y_{ik}\leq 1,\forall k\in K$$ (10)

Constraint (7) stipulates that each EV is dispatched at most once. By enforcing this restriction, the same EV is not redundantly assigned to multiple delivery routes, thereby exerting an indirect effect on the costs associated with vehicle dispatch (*P*_1_). Constraint (8) necessitates that each client is visited uniquely once. In the context of the objective function, this constraint aids in minimizing the travel and service costs (*P*_2_ and *P*_3_), as it discourages unnecessary detours or repeat visits. Constraint (9) enforces flow conservation among customer locations, battery swapping stations, and charging stations. This constraint indirectly impacts energy costs (*P*_4_) by influencing the distribution of EVs and their energy replenishment. Constraint (10) states that each EV is allowed to be recharged at most once on the route. Regarding the objective function, this constraint directly affects the energy cost component (*P*_4_) by placing a cap on recharging events, which can influence the allocation of EVs and their corresponding energy consumption patterns.


$$\sum_{i\in C}D_{i}w_{ik}\leq W,\forall k\in K$$ (11)

$$\lambda_{ik}^{2}=\lambda_{ik}^{1}-D_{i},\forall i\in C,k\in K$$ (12)

Constraint (11) asserts that the duty payload apportioned to an individual EV must not transgress its supreme capacity. From the perspective of the objective function, this constraint influences the travel and energy costs (*P*_2_ and *P*_4_) by constraining the goods that each EV can carry. Inefficient or overloaded routes that could potentially inflate costs are avoided. Constraint (12) states that the load of an EV after serving a client is determined by subtracting the client’s demand from the load before service. This constraint directly impacts the customer service cost (*P*_3_), as it influences the load adjustments after each service operation.


$$S_{0k}^{2}=Q,\forall k\in K$$ (13)

$$S_{ik}^{1}=S_{ik}^{2},\forall i\in C,\forall k\in K$$ (14)

$$S_{jk}^{1}\leq (S_{ik}^{2}-E_{ijk})x_{ijk}+Q(1-x_{ijk}),\forall i,j\in V,\forall k\in K,i\neq j$$ (15)

$$S_{ik}^{1}\geq 0,\forall i\in V,\forall k\in K$$ (16)

$$q_{ik}=Q-S_{ik}^{1},\forall i\in F,\forall k\in K$$ (17)

$$S_{ik}^{2}=Q,\forall i\in B,\forall k\in K$$ (18)

Constraint (13) mandates every EV to be completely charged prior to its departure from the depot. This constraint directly affects the energy cost (*P*_4_), as it ensures that the initial energy state of each EV is at its maximum capacity, thus minimizing the need for immediate recharging or swapping. Constraint (14) expounds that the EV battery level remains unchanged at customer points. This constraint impacts the energy cost (*P*_4_) by maintaining a consistent energy level throughout the service process, which in turn affects the EV’s overall energy consumption. Constraint (15) upholds the energy equilibrium of each EV during its transit from the preceding node *i* to the subsequent node *j*, considering the energy consumption along the arc (*i*,*j*). This constraint directly influences the energy cost (*P*_4_) by accounting for the energy consumed during travel and distribution tasks. Constraint (16) ensures that EV’s remaining energy is not negative when it reaches any node. This constraint affects the feasibility of the solution and indirectly impacts the EV dispatching cost (*P*_1_) by preventing scenarios where an EV’s energy level becomes insufficient for completing the assigned tasks. Constraint (17) defines the amount of charging at a charging station, which directly affects the energy cost (*P*_4_) by determining the energy replenishment needed during the route. Constraint (18) indicates that when the EV departs from the battery swapping station, its battery level becomes full. This constraint impacts the energy cost (*P*_4_) by ensuring that the EV commences its journey with a fully charged battery after swapping.


$$t_{ik}^{2}=t_{ik}^{1}+s_{i}w_{ik},\forall i\in C,\forall k\in K$$ (19)

$$t_{jk}^{1}\leq (t_{ik}^{2}+T_{ijk})x_{ijk}+(1-x_{ijk})M,\forall i,j\in V,\forall k\in K,i\neq j$$ (20)

$$\sigma_{ik}=60\cdot \frac{q_{ik}}{\eta \cdot p_{e}},\forall i\in F,\forall k\in K$$ (21)

$$t_{ik}^{2}=t_{ik}^{1}+\sigma_{ik}y_{ik}+\varphi z_{ik},\forall i\in F\cup B,\forall k\in K$$ (22)

Constraint (19) embodies the correlation amongst the EV’s arrival time, service duration, and departure time at a customer vertex. This constraint affects both the travel cost (*P*_2_) and the customer service cost (*P*_3_) in the objective function. Constraint (20) signifies the temporal interdependence between the EV’s exit time from the present node *i* and its entry time at the ensuing node *j*. This constraint impacts the travel cost (*P*_2_) within the objective function, as it makes the EV’s route go through different traffic conditions. Constraint (21) calculates EV’s charging duration at a charging station. This constraint directly affects the energy cost (*P*_4_) within the objective function, as it influences the charging-related expenses incurred during the distribution process. Constraint (22) signifies the time relationship before and after the EV replenishes energy at a charging station or battery swapping station. This constraint impacts the objective function by regulating the timing of energy replenishment, thereby affecting both the energy cost (*P*_4_) and the overall distribution efficiency.


$$x_{ijk},w_{ik},y_{ik},z_{ik}\in \{0,1\}$$ (23)

Constraint (23), a binary constraint, introduces a discrete decision-making element into the optimization framework. The binary constraint adds a layer of restriction to the optimization process, as the decision to activate or deactivate certain components directly affects the distribution costs captured by the objective function. Depending on the problem specifics, constraint (23) might influence the allocation of vehicles to particular routes, the utilization of charging or battery swapping stations, or the selection of specific distribution tasks.

## 5. Algorithm design

The ALNS metaheuristic was originally introduced by Pisinger and Ropke [66] and has since been adapted to various optimization problems. For instance, Koch et al. [67] employed an ALNS framework for an integrated vehicle routing problem with complex loading constraints involving pickups and deliveries. In a similar vein, Žulj et al. [68] devised a hybrid ALNS that integrates tabu search components for tackling an order batching problem. Notably, Seydanlou et al. [69] tailored a multi-neighborhood search algorithm to address sustainable closed-loop supply chain management in Iran’s agricultural sector. Moreover, Fathollahi-Fard et al. [70] applied an ALNS approach to the generalized quadratic assignment problem and introduced an efficient Benders reformulation based on reformulation linearization technique inequalities. Given this versatility, the ALNS metaheuristic presents a promising foundation for the TDOEVRP-HERS. Expanding upon this foundation, the current framework is extended into a HALNS, drawing inspiration from the ACO metaheuristic. Subsection 5.1 elaborates on the general structure of the HALNS metaheuristic, followed by Subsections 5.2 to 5.5, which provide detailed insights into the distinct constituents of the complete HALNS algorithm.

### 5.1. General framework of HALNS

**Algorithm 1.** The pseudocode of HALNS

1: ACO generates initial solution *initialSol*

2: Global optimal solution *bestSol* ← *initialSol*

3: Current solution *currentSol* ← *initialSol*

4: **while** the termination condition is not met **do**

5: Use roulette rules to choose the destruction operator and repair operator.

6: Use the destruction operator on *currentSol*, and then use the repair operator to repair it, and get an updated solution *updateSol*.

7: **if** *updateSol* is better than *bestSol* **then**

8:  *bestSol* ← *updateSol*

9:  *currentSol* ← *updateSol*

10: **else if** *updateSol* is better than *currentSol* **then**

11:  *currentSol* ← *updateSol*

12: **else**

13:  **if** simulated annealing criterion acceptance **then**

14:   *currentSol* ← *updateSol*

15:  **end if**

16: **end if**

17: Update the scores of destruction operators and repair operators.

18: **if** the number of iterations to update operator weights has been reached **then**

19:  Update the weights of destruction operators and repair operators, and reset the score to zero.

20: **end if**

21: **end while**

The proposed HALNS ingeniously integrates the ALNS technique with the ACO method, synergizing their respective strengths. The ALNS, with its capability for intensive local search, is coupled with the ACO’s global exploration potential. This fusion is achieved by initializing the solution process using ACO’s probabilistic approach, generating diverse initial solutions that serve as a foundation for ALNS to perform more refined local searches. The adaptability of ALNS complements the ACO’s explorative prowess, enabling it to fine-tune solutions and guide the search towards promising regions of the solution space. This harmonized approach leverages the ALNS to enhance the solutions found by ACO, ultimately contributing to improved convergence and solution quality. The implementation framework of HALNS is meticulously outlined in Algorithm 1. The process begins by designing an ACO to generate a higher-quality initial solution. Next, five destruction operators—Random Removal (RdR), Basic Worst Removal (BWR), Related Removal (RlR), Single Point Removal (SPR), and Station-based Removal (SbR)—are introduced. In addition, three repair operators are included: Improved Random Insertion (IRdI), Improved Greedy Insertion (IGI), and Improved Regret Insertion (IRgI). During the ALNS iteration, these operators are randomly selected to work on the current solution. Simultaneously, an appropriate acceptance criterion is established to adaptively adjust the operators’ weights and update the current and optimal solutions. The termination conditions for the algorithm may include the maximum number of iterations or the longest runtime.

### 5.2. The construction of the initial solution

The quality of the initial solution significantly influences the ensuing optimization of the ALNS, and a superior initial solution increases the likelihood of achieving a satisfactory solution. Given the characteristics of the TDOEVRP-HERS, the study employs the ACO to procure an improved initial solution. First, all ants are allocated to depot 0. Each ant then embarks on its journey. Based on the transition probability Formula (24), ants employ a roulette-wheel selection process to choose the customer nodes they visit. These selected customer points are added sequentially to the current route until the ants are no longer eligible to continue customer visits. At this point, the ants are reset to depot 0 and initiate their journey anew. This operation is repeated until all customers in the area have been visited by the ants.


$$P_{ij}^{m}=\{\begin{array}{cc} \frac{{\left[\tau_{ij}\right]}^{\beta_{1}}{\left[\vartheta_{ij}\right]}^{\beta_{2}}}{\sum_{s\in unvisit_{m}}\left({\left[\tau_{is}\right]}^{\beta_{1}}{\left[\vartheta_{is}\right]}^{\beta_{2}}\right)}, & j\in unvisit_{m}\\ 0, & j\in visited_{m} \end{array}$$ (24)

where $P_{ij}^{m}$ is the transition probability for ant *m* to travel from node *i* to node *j*. *unvisit*_*m*_ is the set of customer nodes that ant *m* has yet to visit. *visited*_*m*_ is the set of customer nodes that ant *m* has already visited. *τ*_*ij*_ is the pheromone heuristic parameter, and *ϑ*_*ij*_ is the expectation heuristic parameter, *ϑ*_*ij*_ = 1/*d*_*ij*_. *β*_1_ and *β*_2_ are the weights for pheromone and expectation heuristic factors, respectively.

Once all ants have completed visiting all customers, a global pheromone update is executed on the current travel plan, comprising all ants, as per Formula (25).

$$\tau_{ij}^{new}=\tau_{ij}^{old}(1-rho)+\sum_{m=1}^{M}\Delta \tau_{ij}^{m}$$ (25)

$$\Delta \tau_{ij}^{m}=\{\begin{array}{cc} f/Distance_{m}, & \mathrm{if}\;\mathrm{ant}\;m\;\mathrm{travel}\;\mathrm{through}\;\mathrm{arc}\;(i,j)\\ 0, & \mathrm{otherwise} \end{array}$$ (26)

where $\tau_{ij}^{old}$ is the pheromone before update, while $\tau_{ij}^{new}$ is the updated pheromone. *rho* is the pheromone evaporation rate with 0≤*rho*<1. Set *rho* = 0.2. $\Delta \tau_{ij}^{m}$ signifies the pheromone increment on arc (*i*,*j*) for ant *m*. *f* is a constant that represents the amount of pheromone secreted by an ant in each travel. Set *f* = 5. *Distance*_*m*_ is the total distance traveled by ant *m*.

The specific steps of the ACO are as follows:

Step 1: Parameter initialization. Begin by inputting the necessary test data, which include the location coordinates of all nodes in the transport network, customer delivery volume, EV’s maximum load and battery capacity, congestion time, and time-dependent vehicle speed. Let *Iter*_*Ant*_ represent the current iteration, set *Iter*_*Ant*_ = 1. Let *maxIter*_*Ant*_ represent the maximum number of iterations, set *maxIter*_*Ant*_ = 200. Let *minLen* represent the shortest distance among all ants’ travel, set *minLen* = *Inf*. Let *antNum* represent the number of ants, set *antNum* = 30. Let *initialRoute* represent the initial optimal travel route.

Step 2: Determine whether the *Iter*_*Ant*_ satisfies the relationship of *Iter*_*Ant*_≤*maxIter*_*Ant*_. If it satisfies this condition, assign ant *m* with *m* = 1, and proceed to Step 3 (Here, *tabu*_*m*_ is the ant *m*‘s taboo list, and *tabu*_*m*_ = ∅); otherwise, advance to Step 11.

Step 3: Determine whether *m* satisfies *m*≤*antNum*, and if so, enter Step 4; otherwise, skip to Step 9.

Step 4: Place ant *m* in depot 0 and assign it the maximum allowable load.

Step 5: Based on the transition probability (Formula 24), select a customer node that fulfills the constraints and add it into the current solution. Then, remove this node from ant *m*‘s *unvisit*_*m*_ set, and place it in ant *m*‘s *tabu*_*m*_ set.

Step 6: Repeat the operation of Step 5 until ant *m* no longer meets the requirements to visit any of the remaining customer nodes.

Step 7: Reposition ant *m* to depot 0 and reset ant *m*‘s maximum load. Determine whether the *unvisit*_*m*_ is an empty set. If *unvisit*_*m*_ ≠ ∅, revert to Step 5; otherwise, save ant *m*‘s total travel distance *antLen*_*m*_ to the *iterLen*_*iter*_ set, and save ant *m*‘s travel routes *antSol*_*m*_ to the *iterSol*_*iter*_ set, proceeding subsequently to Step 8.

Step 8: Let *m* = *m*+1, clear the taboo list *tabu*_*m*_, reset the *unvisit*_*m*_ set, and then proceed to Step 3.

Step 9: Analyze all travel distances within the *iterLen*_*iter*_ set to derive the optimal travel distance *bestLen*_*iter*_ and the optimal travel routes *bestRoute*_*iter*_. If *bestLen*_*iter*_≤*minLen*, set *minLen* = *bestLen*_*iter*_ and update the globally optimal routes as *initialRoute* = *bestRoute*_*iter*_. Conversely, if *bestLen*_*iter*_>*minLen*, then set *bestLen*_*iter*_ = *minLen* and *bestRoute*_*iter*_ = *initialRoute*. Subsequently, pheromones along the route are updated following Formula (25) before progressing to Step 10.

Step 10: Let *Iter*_*Ant*_ = *Iter*_*Ant*_+1, return Step 2.

Step 11: Following the principle of greed, insert the charging station or battery swapping station into the optimal initial route, *initialRoute*, according to the battery level constraints. This process culminates in the acquisition of the initial solution, *initialSol*, tailored for ALNS.

### 5.3. Operator design

**Fig 2:**
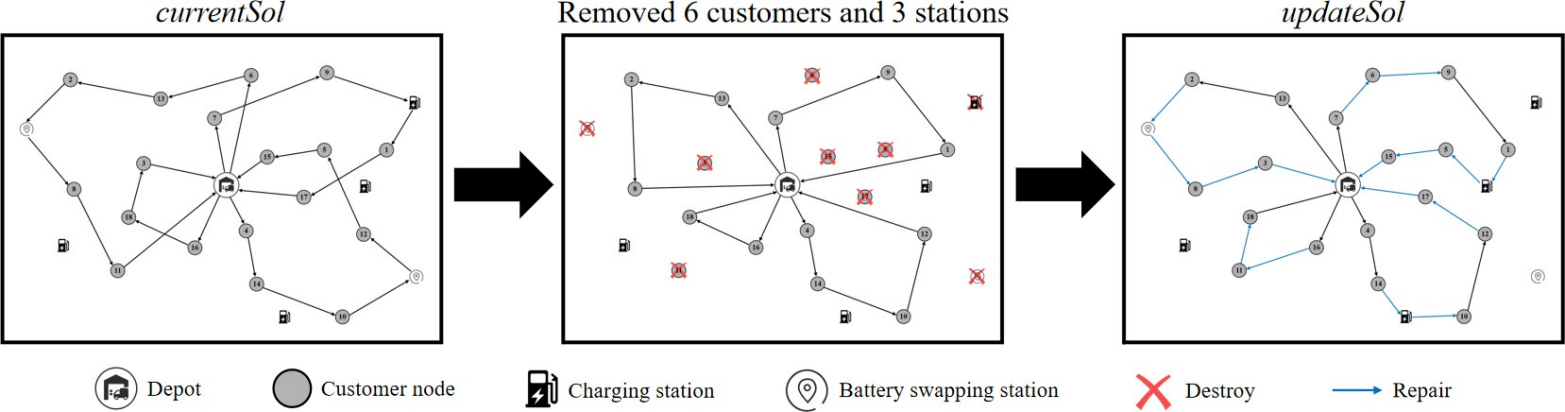
Schematic of the route destruction and repair.

In this study, a comprehensive set of destruction and repair operators are employed within the HALNS. In addition to the fundamental RdR, BWR, and RlR operators, the algorithm introduces two novel operators, SPR and SbR, within the destruction phase, thereby contributing originality to the approach. Moreover, to enhance the algorithm’s performance, three improved repair operators—IRdI, IGI, and IRgI—are adapted from the existing literature. This judicious combination facilitates the efficient restoration of solutions. The sequence of operations unfolds as follows: five destruction operators—RdR, BWR, RlR, SPR, and SbR—disassemble the *currentSol*, which is then reconstructed using the repair operators—IRdI, IGI, and IRgI. Throughout this iterative process, redundant charging or battery swapping stations within *currentSol* are methodically removed, and new energy replenishment strategies are reintegrated during the repair phase to address route infeasibility. For a visual depiction, refer to Fig 2, where the leftmost image presents *currentSol*, the central image demonstrates the outcome of applying the destruction operator (resulting in the removal of six customers and three energy replenishment stations), and the rightmost image showcases *updateSol* following the repair operator’s intervention.

#### 5.3.1. Destruction operator

The destruction operator selectively removes a predetermined number of customer nodes from the current solution. Given a total customer count, *n*, the quantity *r* designated for removal is determined by the equation *r* = [*α*×*n*]. In this equation, *α* represents the removal ratio, and the [] is the rounding symbol. Set *α* = 0.1. The customers removed through this operation are subsequently added to the unvisited customer set, *C*_*unvisit*_, while any invalid routes and superfluous charging or battery swapping stations are discarded. The general structure of destruction operators is offered in Algorithm 2.

**Algorithm 2.** The general framework of destruction operators

**input:** A *currentSol* and the number of requests to be removed *r*

**output:** A *partial*_*solution*

1: Initialize removal list (*C*_*unvisit*_ ← ∅)

2: Use roulette rules to randomly choose a destroy heuristic based on their weights

3: **while** termination criteria are not met **do**

4: Apply the selected destruction operator to remove a request *r*

5: *C*_*unvisit*_ ← *C*_*unvisit*_∪{*r*}

6: **end while**

(1) RdR: Randomly remove *r* customer nodes from the current solution. Algorithm 3 illustrates the implementation framework of the RdR operator.

**Algorithm 3.** The general framework of the RdR operator

1: Initialize *i* ← 0, *C*_*unvisit*_ ← ∅

2: *partial*_*solution* ← *currentSol*

3: **while** *i*<*r* **do**

4: Randomly choose a route *path*_*D*_ to destroy, and randomly remove a customer node *o* from *path*_*D*_.

5:  *C*_*unvisit*_ ← *C*_*unvisit*_∪{*o*}

6:  If *path*_*D*_ has no customer nodes, delete *path*_*D*_.

7:  *i*++

8: **end while**

9: Update *partial*_*solution*, i.e., remove nodes within *C*_*unvisit*_ from *currentSol*.

10: Remove redundant charging or battery swapping stations within *partial*_*solution*.

(2) BWR: The cost of a customer node *i* in the current solution is denoted as *cost*(*s*,*i*) = *f*(*s*)−*f*_−*i*_(*s*). Here, *f*(*s*) represents the objective value of the current solution, and *f*_−*i*_(*s*) is the objective value after removing customer node *i*. Initially, BWR calculates the objective value of the current solution. Subsequently, it computes the objective values after the removal of different customer nodes. After that, it derives the cost associated with each customer node. Finally, it selects the customer node with the highest cost for removal and repeats above step until *r* customer nodes have been removed. This strategy was proposed by Hemmelmayr et al. [71]. Algorithm 4 illustrates the implementation framework of the BWR operator.

**Algorithm 4.** The general framework of the BWR operator

1: Initialize removal list (*C*_*unvisit*_ ← ∅)

2: *partial*_*solution* ← *currentSol*

3: Compute *cost*(*s*,*i*) for all customers *i*∈*C*.

4: **while |***C*_*unvisit*_**|<*r* do**

5: Select the customer node *o* with the highest *cost*(*s*,*i*) for removal.

6: *C* ← *C*\{*o*}, *C*_*unvisit*_ ← *C*_*unvisit*_∪{*o*}

7: Compute *cost*(*s*,*i*) for all customers *i*∈*C*.

8: **end while**

9: Update *partial*_*solution*, i.e., remove nodes within *C*_*unvisit*_ from *currentSol*.

10: Remove redundant charging or battery swapping stations within *partial*_*solution*.

(3) RlR: Initially, a customer node *i* is randomly selected for removal and placed in the *C*_*unvisit*_ set. The similarity function is then used to calculate the similarity between each customer node in the current solution and the removed node. The node with the highest similarity in the current solution is selected for removal and added to the *C*_*unvisit*_ set. Subsequently, a customer node is randomly selected from *C*_*unvisit*_, and the similarity calculation process is repeated, leading to the removal of the node with the highest similarity. This step is repeated until *r* customer nodes have been removed. The similarity function used in this paper is defined as follows:

$$Iden(i,j)=\alpha_{1}d_{ij}+\alpha_{2}|D_{i}-D_{j}|+\eta_{ij}$$ (27)

where *α*_1_ and *α*_2_ represent weight coefficients satisfying the condition *α*_1_+*α*_2_ = 1. Set *α*_1_ = 0.6, *α*_2_ = 0.4. *d*_*ij*_ signifies the distance between two customer nodes, and |*D*_*i*_−*D*_*j*_| represents the difference in delivery volume between two nodes. If nodes *i* and *j* are on the same route, then *η*_*ij*_ = 5; otherwise, *η*_*ij*_ = 0.

(4) SPR: SPR is an approach that involves the defined areas between two energy replenishment stations, or between a replenishment station and a depot, known as a station service area. This process initiates by randomly selecting a service area and a customer node within that area as an operational point. Subsequently, all customer nodes either to the left or right of the operational point within the service area are deleted. The number of removed nodes is counted as *r*_*d*_, leaving a remaining removal capacity, denoted as *r*_*a*_, where *r*_*d*_≤*r* and *r*_*a*_ = *r*−*r*_*d*_. If *r*_*a*_>0, another station service area is randomly selected and the same removal operation is performed, with a simultaneous update of *r*_*a*_. In the final round of the removal operation, if a situation where *r*_*d*_>*r*_*a*_ arises, *r*_*a*_ nodes are randomly chosen for removal from the set of *r*_*d*_ nodes. Algorithm 5 illustrates the implementation framework of the SPR operator.

**Algorithm 5.** The general framework of the SPR operator

1: Initialize removal list (*C*_*unvisit*_ ← ∅)

2: *partial*_*solution* ← *currentSol*, *r*_*a*_ ← *r*

3: Count all service areas and put them into the set *Serve*.

4: **while** *r*_*a*_>0 **do**

5: Randomly select a station service area *i*∈*Serve*.

6: Randomly select a customer node *o* within service area *i*.

7: All *m* customer nodes either to the left or right of node *o* within service area *i* are deleted.

8: *r*_*d*_ ← *m*, *C*_*unvisit*_ ← *C*_*unvisit*_∪*m*, *Serve* ← *Serve*\{*i*}

9: **if** *r*_*d*_>*r*_*a*_

10:  *r*_*a*_ nodes are randomly chosen for removal from the set of *r*_*d*_ nodes.

11:  *r*_*a*_ ← 0

12: **end if**

13: *r*_*a*_ ← *r*_*a*_−*m*

14: **end while**

15: Update *partial*_*solution*, i.e., remove nodes within *C*_*unvisit*_ from *currentSol*.

16: Remove redundant charging or battery swapping stations within *partial*_*solution*.

(5) SbR: SbR begins with the random selection of either a charging station or a battery-swapping station. From the route associated with this selected station, the nearest *m* customer nodes are removed, where *m* can be divisible by *r*. This procedure is repeated until *r* customer nodes have been removed. The study sets *m* = 10. Algorithm 6 illustrates the implementation framework of the SbR operator.

**Algorithm 6.** The general framework of the SbR operator

1: Initialize *m* ← 10, *C*_*unvisit*_ ← ∅

2: *partial*_*solution* ← *currentSol*

3: **while** |*C*_*unvisit*_|<*r* **do**

4: Randomly select a station *i*∈*F*∪*B*.

5: Get routes associated with this selected station *i*.

6: Find the nearest *m* customer nodes on associated routes to station *i* and remove them.

7: *C*_*unvisit*_ ← *C*_*unvisit*_∪*m*

8: **end while**

9: Update *partial*_*solution*, i.e., remove nodes within *C*_*unvisit*_ from *currentSol*.

10: Remove redundant charging or battery swapping stations within *partial*_*solution*.

#### 5.3.2. Repair operator

Given that the basic random repair operator, greedy repair operator, and regret repair operator do not account for the impact of customer node re-insertion on route feasibility, this paper proposes three advanced repair operators: IRdI, IGI, and IRgI. Each repair operator involves a two-stage restoration process. The first stage focuses on ensuring the feasibility of customer delivery routes, while the second stage attends to the feasibility of vehicle energy consumption.

(1) IRdI: From the removed customer set *C*_*unvisit*_, randomly select one customer and identify all permissible insertion locations based on load constraints. Subsequently, an insertable position is randomly selected for customer insertion. Repeat this step until all *r* customers have been reintegrated into the solution. Subsequently, routes that fail to meet power constraints, as determined by time-varying factors and energy consumption estimates, undergo repair via the insertion of energy replenishment stations. Algorithm 7 illustrates the implementation framework of the IRdI operator.

**Algorithm 7.** The general framework of the IRdI operator

1: Initialize *reintegrated*_*count* ← 0

2: **while** *reintegrated*_*count*<*r* **do**

3: Randomly select a customer node o∈*C*_*unvisit*_.

4: Identify locations where node *o* allows insertion.

5: Randomly select a permissible position to insert into *partial*_*solution*.

6: *reintegrated*_*count* = *reintegrated*_*count*+1.

7: *updateSol* ←*partial*_*solution*, *C*_*unvisit*_ ← *C*_*unvisit*_*\*{*o*}

8: **end while**

9: Ensure that *updateSol* meets time-dependent energy consumption constraints by potentially inserting energy replenishment stations.

(2) IGI: Initiate by randomly selecting one customer from the set of removed customers, *C*_*unvisit*_. Identify all feasible insertion positions for this customer, considering load constraints. Subsequently, compute the objective value addition resulting from the insertion of the customer at these potential locations. Choose the location that contributes the least added value for customer insertion. Continue this procedure until all removed *r* customers have been reintegrated into the solution. Afterward, assess the routes considering time-dependent factors and energy consumption estimates, and incorporate energy-supplementary stations into routes that do not satisfy power constraints for the necessary adjustments. Algorithm 8 illustrates the implementation framework of the IGI operator.
(3) IRgI: Initially, identify all possible insertion locations for the removed *r* customers, in accordance with load constraints. Next, calculate the additional objective value incurred by inserting customers at different feasible positions. Define customer *i*‘s regret value, *Re*_*i*_, as $Re_{i}=VC_{i}^{1}-VC_{i}^{2}$, where $VC_{i}^{1}$ represents the least objective value added post customer *i* insertion, and $VC_{i}^{2}$ signifies the second smallest additional objective value following the insertion of customer *i*. Compare the regret values of the removed *r* customers, and choose the one with the largest regret value for insertion at the location with the least objective value increased. Repeat this process until *r* customers have been reintroduced into the solution. Ultimately, accommodate any routes failing to meet power constraints by incorporating charging or battery swapping stations, drawing upon time-varying considerations and energy consumption estimates for guidance.

**Algorithm 8.** The general framework of the IGI operator

1: Initialize *reintegrated*_*count* ← 0

2: **while** *reintegrated*_*count*<*r* **do**

3: Randomly select a customer node *o*∈*C*_*unvisit*_.

4: Identify set *feasible*_*locations* where node *o* allows insertion.

5: *best*_*location* ← *NULL*, *least*_*added*_*value* ← *INFINITY*

6: **for** each *position*∈*feasible*_*locations* **do**

7:  Calculate *added*_*value* in the objective function when node *o* is inserted at *position*.

8:  **if** *added_value*<*least*_*added*_*value* **then**

9:   *least*_*added*_*value* ← *added*_*value*, *best*_*location* ← *position*

10:  **end if**

11: **end for**

12: *reintegrated*_*count* = *reintegrated*_*count*+1.

13: Insert node *o* at *best*_*location*, *updateSol* ← *partial*_*solution*, *C*_*unvisit*_ ← *C*_*unvisit*_\{*o*}

14: **end while**

15: Ensure that *updateSol* meets time-dependent energy consumption constraints by potentially inserting energy replenishment stations.

### 5.4. Acceptance criteria

The acceptance criteria for this research utilize the simulated annealing approach as proposed by Adulyasak et al. [72]. During the iterative process, if the updated solution *updateSol* demonstrates better performance than the current solution *currentSol*, the updated solution is retained. Otherwise, the updated solution is maintained with a probability of $p=e^{-(z(updateSol)-z(currentSol))/T}$. The initial temperature is set to *T*_0_ = 10000 and follows a cooling schedule defined by *T*_*n*_ = *cT*_*n*−1_, where the cooling rate *c* = 0.995. The entire search process concludes once the number of iterations reaches a predefined maximum limit.

### 5.5. The specific realization of the adaptive process

The fundamental principle of ALNS involves the adaptive adjustment of an operator’s weight based on its historical performance. Initially, all operators are assigned equal weight and score, with this study setting the initial operator weight to 10 and the score to 0. Throughout the algorithm’s iterative process, the destruction and repair operators are first selected following the roulette wheel rule. Subsequently, these operators are scored differently based on the quality of the updated solution (*updateSol*) after each iteration. When the number of iterations attains the pre-set threshold, the weights of the operators are updated. As a result, operators with greater weights are more likely to be selected in subsequent iterations.

The search process of ALNS employed in this study consists of *N*_*a*_ stages, each of which must execute *N*_*b*_ iterations. Thus, the maximum number of iterations, *maxIter* = *N*_*a*_⋅*N*_*b*_. Set *N*_*a*_ = 4, *N*_*b*_ = 50, i.e., *maxIter* = 200. Assume the weight of operator *i* in stage *j* as *ω*_*ij*_ and the usage probability of operator *i* in stage *j* as *p*_*ij*_. Then, under the current weight, *p*_*ij*_ can be calculated by the formula $p_{ij}=\omega_{ij}/\sum_{h\in H}\omega_{hj}$, where *H* represents the set of operators to which operator *i* belongs. Define the score of operator *i* in stage *j* as *ε*_*ij*_. In this study, four levels of scores are established, and the update rules for *ε*_*ij*_ are as follows:

$$\varepsilon_{ij}=\{\begin{array}{l} \varepsilon_{ij}+50,\;\mathrm{if}\;updateSol\leq bestSol\\ \varepsilon_{ij}+20,\;\mathrm{if}\;bestSol<updateSol\leq currentSol\\ \varepsilon_{ij}+10,\;\mathrm{if}\;currentSol<updateSol,\mathrm{and}updateSol\;\mathrm{is}\;\mathrm{accepted}\\ \varepsilon_{ij},\;\mathrm{otherwise} \end{array}$$ (28)

In the scoring scheme, the corresponding destruction and repair operators accrue points based on the quality of the updated solution relative to both the global optimal and the current solution. Specifically, if the updated solution surpasses the global optimal solution, the operators involved receive an additional 50 points. If the updated solution, while not surpassing the global optimal, improves upon the current solution, the operators gain 20 points. In cases where the updated solution is not an improvement over the current one but is still accepted, the operators are credited with 10 points. Lastly, if the updated solution neither improves upon the current solution nor is accepted, no points are added to the corresponding destruction and repair operators.

The update rules of the operator weights are as follows:

$$\omega_{i(j+1)}=\{\begin{array}{c} \;\omega_{ij},\;\pi_{ij}=0\\ (1-\theta )\omega_{ij}+\theta \frac{\pi_{ij}}{\varepsilon_{ij}},\;\pi_{ij}>0 \end{array}$$ (29)

where *ω*_*i*(*j*+1)_ denotes the weight of operator *i* in stage (*j*+1), *θ* is the weight adjustment coefficient, indicating the importance of historical weight and operator performance when the operator weight is updated. Set *θ* = 0.3. Additionally, *π*_*ij*_ represents the number of times operator *i* is selected during the *N*_*b*_ iterations of stage *j*. Should operator *i* not be utilized during the current stage *j*, its weight remains unchanged in the subsequent stage. Following a weight update, both *ε*_*ij*_ and *π*_*ij*_ are reset to zero.

## 6. Experiments and analysis

This section offers a detailed account of the test works conducted on the proposed model and algorithm. It covers data preparation, algorithm parameter tuning, experimental evaluations of the superiority of the TDOEVRP-HERS and HALNS, and a thorough analysis of the computational experiences with the HALNS.

### 6.1. Data description

Currently, there is no standardized test dataset specifically tailored for TDOEVRP-HERS, and the geographical distribution of customers varies when 3PL fleets undertake transportation tasks for different enterprises. Consequently, this study refines Solomon’s VRP test sets [73], encompassing clustered distribution test sets (C-type), random distribution test sets (R-type), and randomly clustered distribution test sets (RC-type), utilizing these improved test instances as the experimental dataset. Notably, numerous scholars have generated new test instances based on Solomon’s test sets. For example, Schneider et al. [74] employed the Solomon test sets as a benchmark and introduced 21 charging stations into each test instance, thereby creating the EVRP experimental dataset they necessitated.

Building upon the work of Schneider et al., this study further improves the test instances by converting some of the charging station nodes into battery swapping station nodes. Therefore, new test sets tailored for TDOEVRP-HERS are constructed. Each test instance consists of one depot, 100 customers, five charging stations, and five battery swapping stations. The data provided in these test instances include the coordinates of all vertices, as well as the delivery volumes and service durations for each customer.

To meet the test requirements of this paper, the following data are supplemented: (1) Referring to the update frequency of the traffic congestion index in Beijing, set the duration of each time period as *H* = 15 minutes, thereby dividing the whole day into 96 time periods. (2) The earliest working time for the enterprise’s depot is set as 7:00 a.m. During the traffic peak hours of morning (8:00–9:00) and evening (17:30–19:00), the third-party EV fleet operated at a speed of 20 km/h on the road. (3) The EV fleet is assumed to operate with three time-varying speeds during normal time periods. Utilize the remainder function *χ* = mod(*R*,3) to determine these three different velocity values, where *R* represents the *R*-th time period. When *χ* takes the value 1, 2, or 0, respectively, it corresponds to the respective time-varying travel speeds of the EV as 54 km/h, 72 km/h, or 42 km/h. (4) The remaining parameters of the numerical experiment in this paper are set as follows: *ϕ*^*d*^ = 1.184692, *φ*^*d*^ = 1.112434, *g* = 9.8 m/s^2^, *θ*_*ij*_ = 0°, *C*_*r*_ = 0.012, *L* = 3000 kg, *R*_*c*_ = 0.7, *A*_*w*_ = 3.8 m^2^, *ρ* = 1.2041 kg/m^3^, *W* = 800 kg, *Q* = 50 kWh, *η* = 90%, *p*_*e*_ = 60 kW, *φ* = 10 min, *c*_1_ = 120 yuan/vehicle, *c*_2_ = 0.5 yuan/min, *c*_3_ = 0.4 yuan/min, *c*_4_ = 0.7 yuan/min, *c*_5_ = 50 yuan/time.

The prescribed methodologies within the HALNS framework are implemented using the MATLAB R2020b software and performed on a microcomputer, furnished with a 3.60 GHz processor and a memory capacity of 16 GB of RAM.

### 6.2. Parameter tuning

An initial series of experiments is undertaken to fine-tune the parameters of the ALNS metaheuristic, as detailed in Table 2. For this purpose, 27 representative instances, as proposed by Ticha et al. [75], are chosen. These include the SOL instances r101, r105, c103, c104, rc101, and rc105, each with 50 customers, and the NEWLET instances 1, 2, and 3, each comprising 50 customers and 100 nodes. These nine instances are considered for three correlation tiers: No-Correlation (NC), Weak Correlation (WC), and Strong Correlation (SC).

**Table 2:** Parameter values.

Operations	Parameters	Selected value
Related Removal	Weight associated with distance: *α*_1_	0.6
	Weight associated with demand: *α*_2_	0.4
	Route dependency between node *i* and node *j*: *η*_*ij*_	5
Station-based Removal	The number of customers allowed to be removed at a time: *m*	10
Acceptance criteria	Initial temperature: *T*_0_	10000
	Cooling rate: *c*	0.995
Adaptive strategy	Initial operator weights	10
	Get a new global best solution	50
	Get an improved solution	20
	Ger an accepted non-improving solution	10
	Weight adjustment coefficient: *θ*	0.3

Utilizing these instances, the following procedure is embraced. Initially, the parameters of the RlR heuristic are tuned. The ALNS scheme is then implemented, limited to this destruction operator and the IGI operator. Successively, attention is directed to one of the parameters, testing several values for it. For each value, the tuning instances are solved five times; the value that consistently manifests the best average solution quality is ultimately adopted.

For the SbR heuristic, an identical methodology is applied. Other parameters are systematically adjusted in a similar manner, but the comprehensive ALNS scheme is employed rather than relying on individual destruction and repair heuristics.

### 6.3. Experimental evaluation of the proposed model and algorithm

This section aims to validate the superiority of the proposed model and algorithm through five distinct experiments. Each experiment corresponds to a separate subsection and focuses on a specific aspect of the model or algorithm. Two experiments, found in Subsections 6.3.1 and 6.3.5, are dedicated to examining the effectiveness of the HALNS algorithm. Subsection 6.3.1 evaluates the efficiency of the HALNS in solving test sets featuring diverse geographic distributions of customers. Subsection 6.3.5 assesses the HALNS’s robustness to environmental disturbances, with a specific emphasis on its ability to achieve high-quality solutions amidst varying levels of traffic congestion. The other three experiments, detailed in Subsections 6.3.2, 6.3.3, and 6.3.4, are designed to validate the advantages of the TDOEVRP-HERS model. Subsection 6.3.2 investigates whether switching from FVs to EVs indeed leads to a reduction in the overall transportation costs of logistics fleets while also promoting a sustainable urban environment. Subsection 6.3.3 analyzes whether a hybrid energy replenishment strategy outperforms a single replenishment strategy. Finally, Subsection 6.3.4 examines whether employing a 3PL fleet is more economical and environmentally friendly than utilizing a self-operated fleet. In this way, the five experiments comprehensively evaluate the performance of the HALNS algorithm and the TDOEVRP-HERS model, providing valuable insights for practitioners and policymakers.

#### 6.3.1. EV route planning for different test instances

Various test instances, featuring diverse customer geographic distributions, serve to ascertain the feasibility of the HALNS proposed herein. Table 3 shows the test results, where TN signifies the test set name, TC signifies the total distribution cost incurred by the 3PL fleet (in yuan), FC signifies the fixed EV dispatching cost (in yuan), SC signifies the customer service cost (in yuan), DC signifies the vehicle travel cost (in yuan), EC signifies the EV energy replenishment cost (in yuan), EN signifies the number of energy replenishment times for all EVs, DN signifies the number of EVs enabled for distribution, RT signifies the running time of HALNS (in second), and AVE denotes the average value.

**Table 3:** Test results for different test sets.

TN	TC	FC	DC	SC	EC	EN	DN	RT
C101	989.05	360.00	358.57	200.00	70.48	2.00	3.00	238.56
C102	1000.63	360.00	368.44	200.00	72.19	2.00	3.00	211.47
R101	1113.75	240.00	459.87	200.00	213.88	4.00	2.00	258.58
R102	1134.46	240.00	480.70	200.00	213.76	4.00	2.00	270.08
RC101	1156.86	360.00	444.34	200.00	152.52	3.00	3.00	265.48
RC102	1139.44	360.00	424.00	200.00	155.44	3.00	3.00	281.32
C201	1072.75	360.00	415.96	200.00	96.79	2.00	3.00	248.05
C202	1038.80	360.00	406.98	200.00	71.82	2.00	3.00	239.94
R201	1055.75	240.00	443.63	200.00	172.12	4.00	2.00	257.85
R202	1092.29	240.00	458.52	200.00	193.77	4.00	2.00	270.69
RC201	1156.24	360.00	463.43	200.00	132.81	3.00	3.00	258.32
RC202	1168.23	360.00	454.08	200.00	154.15	3.00	3.00	262.41
AVE	1093.19	320.00	431.54	200.00	141.64	3.00	2.67	255.23

The test results depicted in Table 3 reveal that: (1) The fixed EV dispatching costs, travel costs, and customer service costs account for an average of 29.27%, 39.48%, and 18.30% of the total distribution costs, respectively, totaling 87.04%. This finding suggests that vehicle fixed dispatching costs, travel costs, and customer service costs remain the primary factors affecting distribution costs in urban distribution. In practice, when enterprises engage 3PL fleets for urban distribution, they should require the fleet to use EVs with larger capacities to minimize the number of EVs dispatched. At the same time, to avoid increased travel costs due to traffic congestion, logistics fleets should consider the effect of time-varying vehicle speeds on travel time when performing distribution tasks. Furthermore, it is recommended that fleet managers train fleet members to reduce customer service time and improve service efficiency, which can not only reduce the service cost that enterprises have to pay for the fleet, but also improve the competitiveness of the transport fleet. These efforts can ultimately reduce the total distribution cost of the fleet and foster stronger collaborations between enterprises and 3PL fleets. (2) EV energy replenishment costs during distribution account for a range of 6.91% to 19.20% of the total distribution cost, with an average of 12.82%. It is noteworthy that the majority of EVs on distribution routes only require a single energy supplement at most to complete transportation tasks. Thus, using EVs for urban distribution has a negligible effect on distribution efficiency. Compared to traditional FVs, EVs incur lower energy consumption and offer additional advantages such as zero emissions and reduced noise levels. Embracing EVs for urban distribution can aid logistics fleets in reducing energy consumption and gaining cost edges, all while decreasing the environmental impact of transport activities. This aligns with the vision of policymakers who seek to promote green and sustainable development of urban distribution. (3) The HALNS’s running time ranged from a minimum of 211.47 seconds to a maximum of 281.32 seconds, with an average of 255.23 seconds. This result indicates that the HALNS proposed in this study can provide high-quality EV distribution and energy replenishment route planning solutions that meet decision objectives in a reasonable period, demonstrating both high efficiency and feasibility.

**Fig 3:**
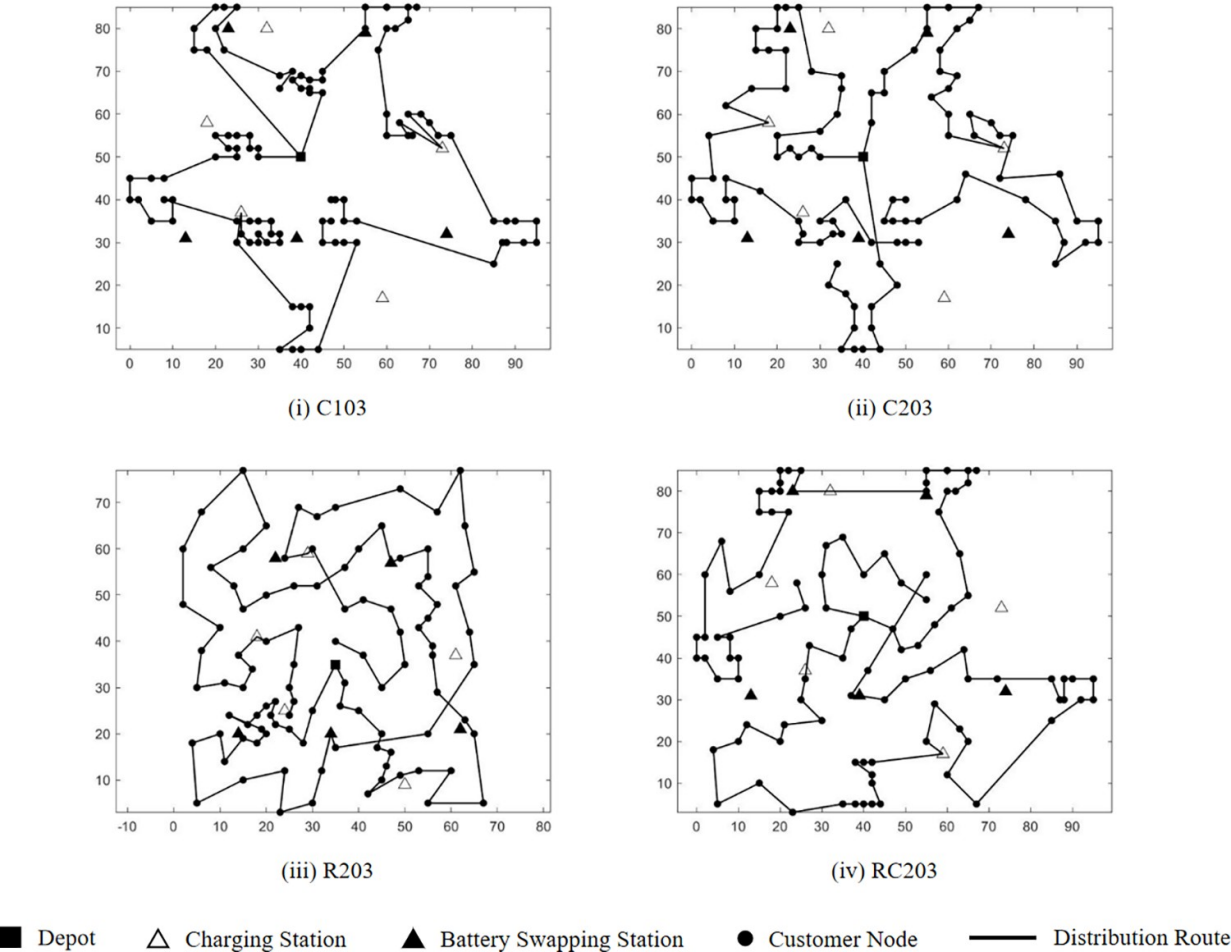
EV route planning solutions for different test instances.

Fig 3 illustrates the EV distribution and energy replenishment route planning solutions for the C103, C203, R103, and RC203 test instances. The solutions for each test instance involving 100 customers are shown clearly and distinctly in the figure, with few instances of route detours and intersections. Based on test cases reflecting a realistic delivery scale, the proposed algorithm demonstrates its potential to consider various practical factors and offer valuable guidance for the transportation route optimization of logistics fleets.

#### 6.3.2. Comparative tests of TDOEVRP and TDOFVRP

A comparative experiment is conducted to analyze the differences in urban distribution costs between the utilization of EVs and FVs. Multi-type test sets are employed while keeping relevant parameters unchanged. While FVs do not account for energy supplement time during distribution, they generate fuel consumption and carbon emissions. Following the methodology and experimental parameters employed by Liu et al. [20] for calculating fuel consumption and carbon emissions of FVs in their VRP study, the associated costs are incorporated into the distribution expenses of the logistics fleet. Table 4 presents the results of comparative experiments between TDOEVRP and TDOFVRP (Time-Dependent Open Fuel Vehicle Routing Problem), where TD signifies the total EV travel distance, GC signifies fuel consumption costs, and CC signifies carbon emission costs incurred by FVs. Remaining symbols maintain their previously established definitions.

**Table 4:** Test results of EV urban distribution and FV urban distribution.

TN	TDOEVRP			TDOFVRP			
	TC	TD	EC	TC	TD	GC	CC
C104	1012.78	559.83	70.67	1506.34	511.98	527.68	13.28
R104	1108.42	714.62	183.14	1607.36	691.12	611.43	15.38
RC104	1132.64	709.86	136.66	1629.58	688.85	599.30	15.08
C204	1067.80	644.38	92.52	1549.24	608.24	541.27	13.62
R204	1100.51	704.67	175.28	1598.73	666.73	594.06	14.95
RC204	1145.95	725.49	153.26	1631.47	701.58	607.68	15.29
AVE	1094.68	676.48	135.26	1587.12	644.75	580.24	14.60

The test results depicted in Table 4 reveal that: (1) Using EVs instead of FVs for urban distribution offers significant cost savings for logistics fleets. Specifically, substituting EVs for FVs leads to an average reduction of 31.03% in distribution costs, with the energy consumption cost for the logistics fleet decreasing from an average of 36.56% to 12.36% of the total distribution cost—a remarkable decline of 66.20%. This finding highlights the advantages of EV fleets in urban distribution, including energy savings and reduced operational expenses. To enhance competitiveness, logistics fleets should adopt EVs for their distribution operations, which aligns with society’s call for sustainable development within the logistics industry. Government departments should promote EV distribution by strengthening the construction and management of EV energy replenishment infrastructures and enhancing the convenience and safety of EV fleet use. (2) Using EVs for urban distribution leads to a mere 4.69% average increase in total distance traveled compared to using FVs. This finding can be attributed to the well-developed energy replenishment infrastructure in urban areas, which makes it easy for EVs to locate nearby charging stations or battery swapping stations. Additionally, using FVs for urban distribution generates an average carbon emission cost of 14.60 yuan, while EVs produce no carbon emissions, improving urban air quality and promoting sustainable logistics transportation. However, it is worth noting that the carbon emission cost of FV distribution accounts for only 0.92% of the total distribution cost, which is less than 1% of the proportion, indicating that logistics fleets do not prioritize reducing emissions when using FVs for urban distribution. This result suggests that China’s current carbon price is too low to incentivize logistics fleets to curtail emissions actively. Therefore, Chinese policymakers should formulate more reasonable carbon trading-related policies to promote sustainable logistics transportation.

#### 6.3.3. Comparative tests of different energy replenishment strategies

A comparative experiment is conducted to assess the effects of various energy replenishment strategies on EV distribution costs, including Hybrid Energy Replenishment Strategy (HERS), Pure Charging Strategy (PCS), and Pure Battery Swapping Strategy (PBSS). The experiment uses multiple types of test sets while keeping the other parameters unchanged. The comparative test results of the three energy replenishment strategies are presented in Table 5, where TT is the total distribution time for all EVs (in minutes), which encompasses EVs’ travel time on the route, customer service time, and energy replenishment time, and ET is the total energy replenishment time for all EVs (in minutes). Remaining symbols maintain their previously established definitions.

**Table 5:** Test results for different energy replenishment strategies.

TN	HERS			PBSS			PCS		
	TC	TT	ET	TC	TT	ET	TC	TT	ET
C105	1007.10	1282.18	65.04	1020.17	1240.33	20.00	991.96	1322.38	103.83
R105	1070.84	1470.82	82.14	1088.34	1436.68	40.00	1001.68	1545.74	194.03
RC105	1141.21	1460.95	73.69	1157.13	1424.26	30.00	1145.04	1602.24	169.58
C205	1083.54	1433.33	69.37	1123.98	1357.96	30.00	1076.64	1483.19	125.23
R205	1087.02	1502.04	85.00	1105.03	1470.05	40.00	1021.88	1588.12	189.08
RC205	1122.05	1422.36	74.36	1140.78	1391.55	30.00	1103.65	1522.42	162.21
AVE	1085.29	1428.61	74.93	1105.90	1386.81	31.67	1056.81	1510.68	157.33

The test results depicted in Table 5 reveal that: (1) In each test instance, the EV energy replenishment time achieved with PBSS is significantly lower than the other two strategies. Compared to HERS, PBSS reduces EV energy supplement time by a range of 51.30% to 69.25%, with an average saving of 58.20%. Compared to PCS, PBSS exhibits even more substantial savings in EV energy supplement time, with an impressive average time-saving of 79.80%. However, it should be noted that PBSS comes with higher distribution costs for logistics fleets. The total distribution costs incurred by PBSS are the highest among all test instances. On average, the use of PBSS increases the distribution cost of the logistics fleet by 4.74% compared to PCS. Therefore, when the 3PL fleet undertakes distribution tasks for enterprises, it is crucial to assess the urgency of customer package delivery. If there are time-sensitive customers, opting for battery swapping stations for EV energy replenishment can enhance delivery efficiency. On the other hand, if customers do not prioritize timeliness, charging stations can be utilized to reduce distribution costs. (2) Although HERS does not offer the optimal solution in terms of both total distribution cost and total distribution time, it exhibits a modest increase in distribution cost of only 2.65% on average compared to PCS, which has the lowest distribution cost. Similarly, compared to PBSS, which achieves the shortest distribution time, HERS shows a slight increase in distribution time by an average of 2.94%. Despite PCS being cost-effective, it requires an average of 5.69% more distribution time than HERS. Likewise, although PBSS reduces distribution time, it incurs an average of 1.90% higher distribution costs than HERS. These findings highlight the ability of HERS to strike a balance between total distribution costs and distribution efficiency, providing flexibility in energy replenishment operations during transportation tasks. Therefore, using HERS to support the urban distribution of EV fleets is a more scientific and reasonable approach.

#### 6.3.4. Comparative tests of open routing and closed routing

A comparative experiment is conducted to analyze the differences in urban distribution route planning between self-operated transport fleets and 3PL fleets. Multi-type test sets are utilized while keeping the relevant parameters unchanged. It should be noted that the self-operated transport fleet follows a closed routing, which requires returning to the enterprise’s depot upon completing the distribution task. Conversely, the 3PL fleet adopts an open routing, eliminating the need to return to the depot after task completion. The test results are presented in Table 6, where TCSR denotes the proportion of total distribution cost saved by hiring a 3PL fleet instead of a self-operated transport fleet (in %), and TDSR denotes the proportion of total distribution distance saved by hiring a 3PL fleet (in %). Remaining symbols maintain their previously established definitions.

**Table 6:** Test results of open routing and closed routing.

TN	Open routing			Closed routing			TCSR	TDSR
	TC	TT	TD	TC	TT	TD		
C106	1002.36	1279.26	562.50	1093.05	1376.03	626.16	8.30%	10.17%
R106	1107.02	1473.55	706.69	1162.77	1545.78	812.59	4.79%	13.03%
RC106	1131.06	1459.17	725.88	1195.33	1544.59	762.46	5.38%	4.80%
C206	1063.31	1438.46	648.26	1136.80	1520.12	704.33	6.46%	7.96%
R206	1119.37	1508.03	708.35	1166.29	1577.66	797.80	4.02%	11.21%
RC206	1142.90	1431.86	735.15	1245.90	1536.31	806.98	8.27%	8.90%
AVE	1094.34	1431.72	681.14	1166.69	1516.75	751.72	6.20%	9.35%

The test results depicted in Table 6 reveal that: (1) Engaging a 3PL fleet leads to an average savings of 6.20% and 5.61% in total distribution cost and time, respectively, compared to deploying a self-operated transport fleet. This advantage stems from the fact that when a third-party EV fleet is hired, EVs are not required to return to the company’s depot after completing the distribution task. As a result, the empty travel time of EVs is reduced, leading to decreased travel costs in logistics transportation. Therefore, for companies with an imperfect warehouse network layout, specifically lacking multiple depots, opting for a third-party EV fleet instead of a self-operated fleet can effectively reduce their operational expenses. (2) Hiring a 3PL fleet leads to a considerable reduction in EV travel distance compared to dispatching a self-operated transport fleet, with an average decrease of 9.35%. This finding highlights the potential for further optimizing EV distribution-energy supplement route planning when employing 3PL fleets, resulting in more efficient distribution solutions for EVs. Therefore, promoting this collaboration model is recommended for practical logistics distribution.

#### 6.3.5. Robustness analysis of HALNS under different traffic conditions

While keeping other experimental parameters constant, five distinct combinations of traffic conditions (Ⅰ, Ⅱ, Ⅲ, Ⅳ, Ⅴ) are designed. These conditions are outlined in detail in Table 7, where STC represents severe traffic congestion during the current time period, MTC represents mild traffic congestion during the current time period, TFS indicates that the traffic flows smoothly during the current time period, and the values provided represent the average travel speeds of EVs under the respective traffic scenarios (in km/h). Employing the RC107 test set for experimentation, the test results are presented in Table 8, where DT denotes the travel time on the routes (in min), and PU denotes the total energy consumption of all transport EVs (in kWh). Remaining symbols maintain their previously established definitions.

**Table 7:** Average speeds of EVs in different traffic conditions.

Traffic scenarios	Different traffic conditions and their time distributions				
	STC	TFS	MTC	TFS	STC
	[07:00–9:00]	[09:00–12:00]	[12:00–14:00]	[14:00–17:00]	[17:00–19:00]
Ⅰ	20	35	30	35	20
Ⅱ	25	45	40	45	25
Ⅲ	30	55	50	55	30
Ⅳ	35	65	60	65	35
Ⅴ	40	75	70	75	40

**Table 8:** Test results of the TDOEVRP-HERS in different traffic scenarios.

Traffic scenarios	TC	TT	DT	EC	EN	PU
Ⅰ	1219.85	1819.70	1319.70	0.00	0.00	107.86
Ⅱ	1214.73	1629.45	1109.45	100.00	2.00	143.74
Ⅲ	1157.57	1490.87	910.23	142.45	3.00	182.47
Ⅳ	1173.58	1430.83	835.01	196.07	5.00	240.88
Ⅴ	1189.12	1369.37	757.19	250.53	6.00	313.07

The test results depicted in Table 8 reveal that: (1) The enhancement of traffic conditions yields a gradual reduction in the travel time of EVs during transit. Transitioning from traffic scenario Ⅰ to scenario Ⅴ, the en-route travel time of EVs diminishes by 42.62%. This finding emphasizes the affirmative significance of ameliorated urban traffic on transport efficiency. (2) While the amelioration of traffic conditions boosts the transportation efficiency of 3PL fleets, it also leads to a notable escalation in energy consumption. Shifting from traffic scenario Ⅰ to scenario Ⅴ, the energy consumption of EVs rises by 190.26%. Heightened energy consumption necessitates more frequent energy supplements. Consequently, EVs fail to achieve the lowest total distribution cost in traffic transport scenario Ⅴ, but instead achieve it in transportation scenario Ⅲ. This is attributed to the fact that the transition from transportation scenario Ⅲ to scenario Ⅴ results in a 75.87% increase in energy replenishment costs, whereas the reduction in travel costs amounts to only 16.81%. The rate of increase in energy costs surpasses the decrease in travel costs. Thus, it becomes apparent that when planning the transportation route for the urban distribution fleet, fleet managers must establish a reasonable travel speed range to strike the optimal balance between efficiency and total cost. (3) In comparison to traffic scenario Ⅲ, which boasts the lowest total distribution cost, the average disparity between the total distribution cost of other traffic scenarios and scenario Ⅲ is 3.46%. This minor gap in distribution costs showcases the adaptability and route optimization capabilities of the HALNS based on real-time traffic conditions and time-dependent constraints. When traffic conditions deteriorate, the proposed HALNS prioritizes the delivery of customers in closest proximity to minimize travel time. Conversely, when traffic conditions improve, the HALNS adeptly schedules the insertion of energy-supplementary stations, effectively striking a balance between energy costs and congestion impacts.

### 6.4. Algorithm comparative test

To assess the efficacy of the proposed HALNS, a series of comparative analyses are performed, spanning two experimental categories: comparisons with an exact solver and other metaheuristics. For small-scale computations, outcomes from the HALNS are juxtaposed with those from the widely-recognized CPLEX solver, while for large-scale computations, HALNS’s optimized results are set against those from two renowned metaheuristics. These benchmarks facilitate a thorough appraisal of the HALNS’s performance across varying scales, underscoring its aptitude and preeminence in addressing large-scale optimization challenges.

#### 6.4.1. Comparison with exact solver

In order to validate the proposed mathematical model and assess the performance of the HALNS algorithm, the CPLEX solver version 12.6 is used to address small-scale test instances. These results are then juxtaposed against those produced by the HALNS. To ensure the generality of the experiment, selected subsets from Goeke’s dataset [76] are utilized, with certain charging station coordinates adjusted to battery-swapping station coordinates to accommodate experimental requirements. Each test instance is run through HALNS 10 times to discern the optimal result. The findings are tabulated in Table 9. Within the table, NC denotes the number of customers, NS indicates the number of charging stations, NB signifies the number of battery swapping stations, and ‘gap’ highlights the gap between the ALNS-derived solution and that from CPLEX. Remaining symbols maintain their previously established definitions.

**Table 9:** Small-scale experimental results.

TN	NC	NS	NB	CPLEX		HALNS		gap
				TC	RT	TC	RT	
c101C5	5	2	1	252.58	20.65	252.58	0.61	0.00
c101C10	10	3	2	400.09	88.39	400.09	1.43	0.00
c103C15	15	3	2	414.42	201.72	414.42	4.49	0.00
c202C10	10	4	1	375.64	82.74	375.64	1.36	0.00
c202C15	15	4	1	522.65	196.43	522.65	5.28	0.00
r102C10	10	2	2	387.24	81.98	387.24	1.53	0.00
r105C5	5	2	1	209.23	19.78	209.23	0.83	0.00
r201C10	10	3	1	285.65	86.33	285.65	1.47	0.00
r202C15	15	4	2	478.72	217.45	478.72	4.50	0.00
rc102C10	10	2	2	497.03	82.91	497.03	1.42	0.00
rc105C5	5	3	1	244.53	21.43	244.53	0.71	0.00
rc201C10	10	3	1	421.95	85.67	421.95	1.40	0.00
rc202C15	15	3	2	480.59	190.32	480.59	4.34	0.00
AVE	10.38	2.92	1.46	382.33	105.83	382.33	2.26	0.00

Table 9 delineates that the HALNS is adept at efficiently tackling small-scale TDOEVRPs-HERS. Given the lower number of vertices, HALNS consistently identifies exact solutions that coincide with those derived from the CPLEX solver. However, considering the NP-hard nature of TDOEVRP-HERS, the computational duration for CPLEX escalates dramatically with increasing instance size. As a result, CPLEX’s average execution time for all test instances clocks in at 105.83 seconds, in stark contrast to HALNS’s mere average of 2.26 seconds. This observation accentuates HALNS’s proficiency in navigating towards optimal solutions while preserving exceptional efficiency.

#### 6.4.2. Comparison with other metaheuristics

To further validate the superiority of HALNS, an Ant Colony System (ACS) and a Genetic Algorithm (GA) are developed to tackle the TDOEVRP-HERS. The parameter settings of ACS align with the work studied by Li et al. [77], while the parameter settings of GA follow the experimental setup of Park et al. [78]. Multi-type test sets are used to compare the performance of HALNS, ACS, and GA. Table 10 reports the test results, with the symbols retaining their previously defined meanings.

**Table 10:** The results of algorithm comparative test.

TN	HALNS			ACS			GA		
	TC	TD	RT	TC	TD	RT	TC	TD	RT
C108	985.61	560.69	216.20	1003.16	578.41	167.04	1007.47	577.99	178.72
R108	1086.89	706.54	231.47	1135.61	711.59	193.68	1185.61	742.15	196.27
RC108	1137.81	728.62	224.97	1163.36	738.14	185.37	1215.70	742.01	179.10
C208	1065.27	650.32	218.69	1093.36	661.78	179.05	1124.51	667.95	183.98
R208	1103.65	712.40	235.85	1137.62	751.30	201.57	1142.04	754.02	194.99
RC208	1134.01	721.13	225.66	1151.99	739.40	188.31	1155.23	731.91	199.51
C109	993.57	571.43	221.53	1022.23	589.35	169.35	1027.19	591.74	181.63
R109	1079.90	701.45	240.02	1138.55	720.28	206.19	1177.40	739.46	194.53
RC109	1142.96	735.19	217.48	1194.53	764.62	177.56	1217.61	778.53	181.92
C209	1063.46	647.28	211.74	1102.63	670.19	173.26	1122.77	678.86	191.06
R209	1114.37	719.53	227.91	1168.75	767.41	199.68	1167.39	770.05	192.56
RC209	1140.85	732.95	231.79	1191.07	764.03	186.95	1205.46	781.52	209.42
AVE	1087.36	682.29	225.28	1125.24	704.71	185.67	1145.70	713.02	190.31

**Fig 4:**
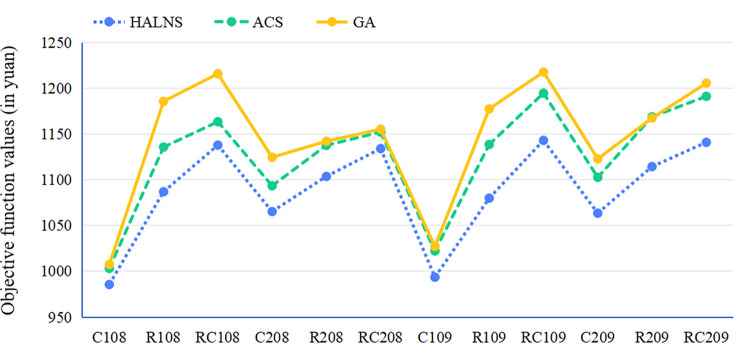
Objective function values of each algorithm for solving large-scale problems.

**Fig 5:**
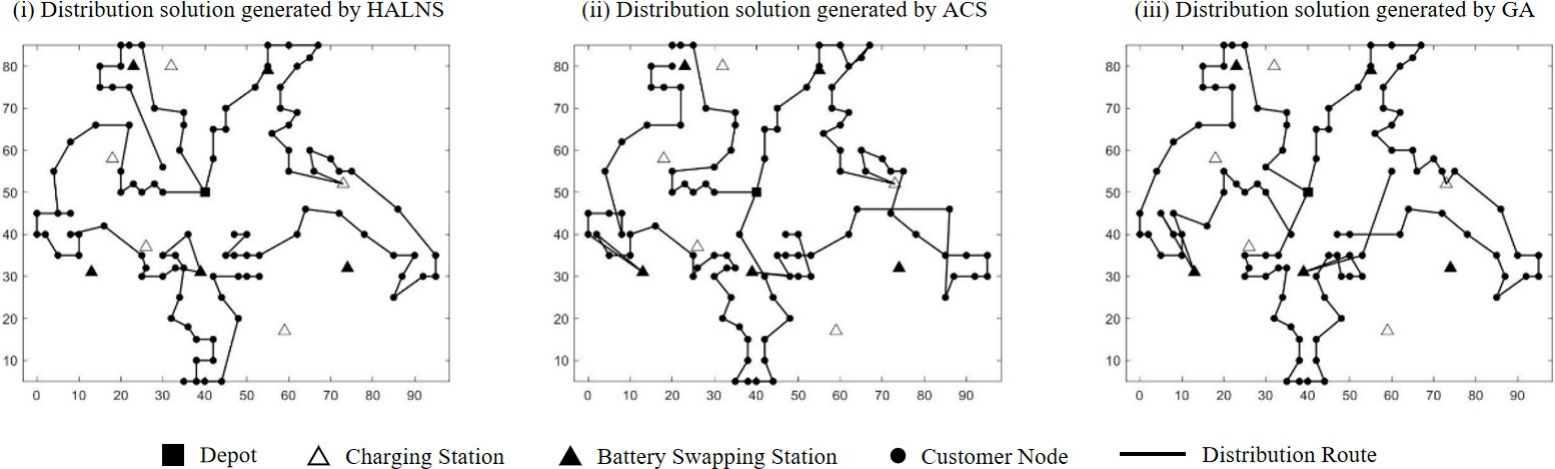
The EV distribution solution of each algorithm.

**Fig 6:**
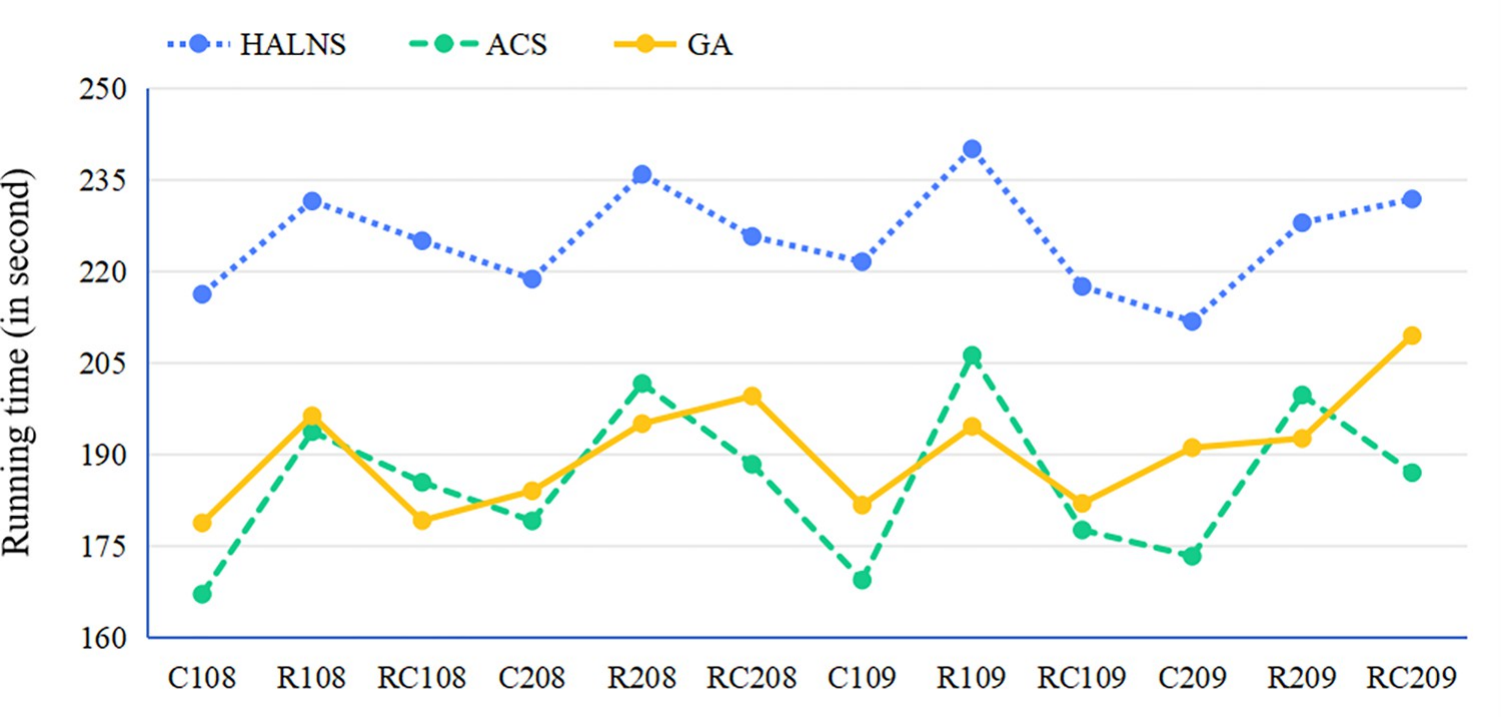
The processing time of each algorithm for solving large-scale problems.

The test results depicted in Table 10 reveal that: (1) In each test instance, HALNS consistently outperforms ACS and GA in terms of total distribution cost and travel distance. On average, HALNS achieves savings of 3.37% in distribution cost and 3.18% in travel distance compared to ACS, and 5.09% in distribution cost and 4.31% in travel distance against GA, respectively. Fig 4 visually illustrates the gaps in the objective function values among the three algorithms, clearly showing HALNS routinely yielding the most favorable objective function values. Meanwhile, Fig 5 showcases the EV distribution route schemes generated by HALNS, ACS, and GA for test set C208. Unmistakably, the HALNS-generated solution delineates the most coherent distribution routes with minimal situations of route detours and intersections. In addition, the energy replenishment stations selected in the HALNS solution are conveniently located near the served customers, while the solutions generated by ACS and GA allocate some energy supplement stations far away from customers to the transport fleet, suggesting that ACS and GA may not reasonably optimize the energy supplement route in transportation, which will affect distribution timeliness and lower customer satisfaction. These findings exhibit the effectiveness of HALNS in optimizing logistics distribution routes, improving logistics distribution efficiency, and reducing logistics distribution costs. (2) Despite the strong optimization capabilities of the HALNS, it demands an average of 17.58% more running time than the ACS and 15.52% more than the GA. Fig 6 highlights the time-consuming calculation burden of the three algorithms through a line graph when processing different test instances. This result can be attributed to three key aspects of the HALNS: operational complexity, depth of search, and adaptivity overhead. Firstly, HALNS integrates multiple destroy and repair heuristics, enabling it to explore a broader solution space and thereby increasing its potential to uncover high-quality solutions. However, the utilization of these operators tends to be computationally taxing, and thus extends the overall processing time. Secondly, the adaptive nature of the HALNS means that it will often perform a more thorough and exhaustive search of the solution space, as compared to ACS or GA. This deeper exploration can yield better results but requires more processing time. Finally, unlike simpler algorithms like ACS or GA, HALNS incorporates an adaptivity mechanism where it modifies the use of different operators based on their performance. This feature enhances the flexibility and robustness of HALNS, but it contributes to the increased running time as the algorithm needs to constantly evaluate and adjust the weights assigned to various heuristics. Consequently, optimizing the solving efficiency of HALNS, without compromising solution quality, will significantly influence its broader application in future contexts.

### 6.5. Sensitivity analysis

**Fig 7:**
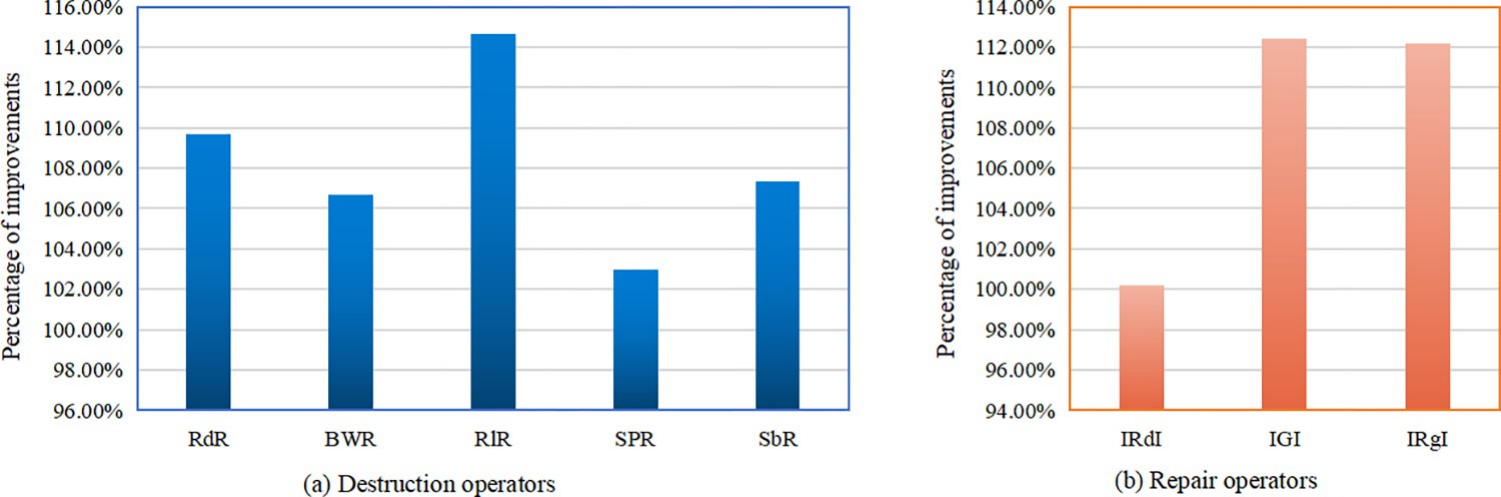
Performance of destruction (a) and repair (b) operators.

Upon validating the superior performance of the proposed HALNS, sensitivity analyses are performed on the destruction and repair operators to assess their efficiency and influence on computational costs. For these evaluations, test set R101 is specifically chosen, and the average changes between the initial and final solutions, as determined by the HALNS algorithm, are calculated. These findings are tabulated in Table 11. To scrutinize the efficacy of each operator, the HALNS is executed using a singular pair of destruction-repair operators, and improvements in the solutions are documented. A subsequent analysis, focusing on the individual performance of the destruction and repair operators, processes the average changes noted in Table 11 for each operator and visually represents them in Fig 7.

**Table 11:** Sensitivity analysis on the performance of destruction and repair operators.

Destruction operators	Repair operators	Relative changes on the solutions by the pair of destruction-repair operators
RdR	IRdI	96%
	IGI	118%
	IRgI	115%
BWR	IRdI	103%
	IGI	110%
	IRgI	107%
RlR	IRdI	104%
	IGI	120%
	IRgI	120%
SPR	IRdI	98%
	IGI	106%
	IRgI	105%
SbR	IRdI	100%
	IGI	108%
	IRgI	114%

Results from Table 11 indicate that the optimal pair of heuristics is the combination of RlR with IGI and IRgI. These two pairings yield an average enhancement of approximately 120% from the initial to the final solution. Upon examining Fig 7, it is evident that RlR serves as the most effective destruction operator (Fig 7(A)), while IGI stands out as the superior repair operator (Fig 7(B)), showcasing a 12.20% overall improvement when compared to IRdI.

In conclusion, the operators employed in the proposed HALNS are pivotal in shaping computational costs. They enhance search ability by adeptly selecting and integrating elements during the solution formulation. By carefully choosing the most fitting destruction and repair operators, the HALNS can traverse the solution space with greater efficacy and attain marked enhancements in solution quality with diminished computational demands.

### 6.6. Discussion

Optimization algorithms have evolved remarkably, fostering advancements across various domains, ranging from online learning, scheduling, and transportation to medicine and data classification. Given the increasing complexity of contemporary decision problems, incorporating advanced algorithms will be instrumental in deriving more effective solutions. The decision problem tackled in this research, the TDOEVRP-HERS, predominantly relied on the HALNS algorithm. While HALNS offers significant merits, it becomes imperative to juxtapose it with other advanced optimization methods to foster comprehensive solution frameworks.

Advanced optimization techniques, such as hybrid heuristics, metaheuristics, hyperheuristics, self-adaptive algorithms, island algorithms, and polyploid algorithms, bring forth specialized capabilities, enabling researchers to address the nuanced facets of complex decision problems. Their adaptability and customizability render them suitable for a plethora of applications. For instance, an online-learning-based evolutionary many-objective algorithm has been manifested to optimize decision-making in dynamic environments [79]. Adaptive polyploid memetic algorithms have demonstrated effectiveness in scheduling applications, such as optimizing truck schedules at cross-docking terminals [80]. In the realm of VRP, algorithms that accommodate both exact and metaheuristic methodologies have been applied, ensuring robust solutions even in multi-objective settings [81].

Given the myriad applications of these algorithms, their relevance to TDOEVRP-HERS cannot be understated. Hybrid meta-heuristic algorithms, for instance, have shown considerable promise in supply chain network designs under uncertain conditions [82], suggesting potential adaptability for electric vehicle routing under time-varying traffic networks. Similarly, the customizability offered by hyper-heuristics can be of paramount importance, especially when devising metaheuristics for continuous optimization [83]. This points towards their potential utility in fine-tuning the solutions for TDOEVRP-HERS.

In conclusion, while the HALNS has proven its merit in this research, the vast landscape of advanced optimization algorithms holds significant promise. By embracing a multifaceted approach that incorporates insights from various algorithms and domains, the research community can pave the way for more affluent, nuanced solutions to intricate decision problems like TDOEVRP-HERS.

## 7. Conclusions

The study investigates the TDOEVRP-HERS as applicable to urban distribution. Therefore, an approach is devised to estimate EV energy consumption within a dynamic urban traffic network. In focusing on third-party logistics distribution, a MIP model is developed with the objective of minimizing the 3PL fleet’s total distribution costs. Based on the model’s characteristics, a HALNS is designed to tackle the model. Comprehensive numerical experiments are conducted to verify the feasibility of the proposed model and algorithm. The key findings of the experiments are as follows:

1. Cost components: Principal determinants affecting transportation fleets’ distribution costs encompass fixed vehicle dispatching, travel expenses, and customer service costs. Employing EVs in urban transit settings does not lead to exorbitant costs. In fact, EVs are more energy-efficient than FVs, enhancing the competitive edge of 3PL fleets. Their low-carbon footprint further accentuates urban logistical transport’s sustainability, mitigating greenhouse gas emissions and conserving the urban environment.
2. 3PL fleets’ flexibility: The capacity of 3PL fleets to provide adaptable distribution solutions negates the necessity to return to the depot post-task, optimizing route planning and reducing travel distances. Leveraging the benefits of energy conservation and low emissions by EVs, they can substantially diminish greenhouse gas emissions, endorsing eco-friendly transportation practices. Especially given the rising inclination towards low-emission zones in cities, collaboration with third-party EV fleets becomes essential for enterprises.
3. Energy supplement strategies: The battery swapping method, while enhancing distribution efficacy, entails higher costs. Conversely, the EV charging approach is cost-effective but time-intensive. The hybrid strategy, though not optimal in terms of cost and time, exhibits minimal cost variance when compared to the charging method and mirrors the battery-swapping technique in distribution time. Recognizing the diverse energy demands of varying routes and schedules, hybrid strategies grant the 3PL fleet the leeway to customize energy top-ups.

Based on the above findings, recommendations emerge. Enterprises, especially those grappling with non-optimized warehousing networks, should actively contemplate synergies with third-party EV fleets. Not only does this present an avenue for operational cost reductions, but it also ushers in a shift towards eco-friendly urban logistics.

While this research offers a robust foundation, it is circumscribed to the dynamics of urban distribution, focusing primarily on 3PL distribution scenarios. Thus, broader applications may require additional nuances. Future directions will investigate the potential applicability and modifications of the HALNS algorithm in other logistical scenarios and delve deeper into how destruction-and-repair operators in various urban settings might improve the route planning of 3PL fleets.

## Supporting information

S1 Appendix (DOC)

S1 Data (RAR)
