# Supplementary material for: A hybrid adaptive large neighborhood search for time-dependent open electric vehicle routing problem with hybrid energy replenishment strategies
Source: PLoS One. 2023 Sep 14;18(9):e0291473. doi: 10.1371/journal.pone.0291473 (PMC10501597; doi:10.1371/journal.pone.0291473)
Supplement: S1 Appendix — (DOC) [file pone.0291473.s001.doc]

**Appendix A. Notations**

The framework of TDOEVRP-HERS, aligning with the standard VRP delineation, is conceptualized through a complete graph $G=(V,A)$, where $V$ denotes the set of $n$ nodes and $A$ signifies the set of arcs as $A=(i,j):0\leq i,j\leq n;\text{ }i\neq j$. In this context, the notations underpinning the formulated model are defined as follows:

**Indices and sets**

| $F$ | Set of charging stations |
| --- | --- |
| $B$ | Set of battery swapping stations |
| $C$ | Set of customer locations |
| $V$ | Set of all vertices in the transportation network, $V\text{=}F\cup B\cup C\cup0$, where $0$ denotes depot |
| $K$ | Set of all EVs, $K=\{1,2,...,k\}$ |

**Parameters**

| $i$ | A single node in the transportation network, $i\in V$ |
| --- | --- |
| $W$ | EV maximal load |
| $Q$ | EV battery capacity |
| $D_{i}$ | Delivery volume required by customer $i$ |
| $s_{i}$ | The duration required to serve customer $i$ |
| $t_{ik}^{1}$ | The time when EV $k$ arrives at node $i$ |
| $S_{ik}^{1}$ | The remaining battery level of EV $k$ upon arriving at node $i$ |
| $\lambda_{ik}^{1}$ | The real-time load of EV $k$ upon arriving at node $i$ |
| $t_{ik}^{2}$ | The time when EV $k$ leaves node $i$ |
| $S_{ik}^{2}$ | The remaining battery level of EV $k$ upon departing from node $i$ |
| $\lambda_{ik}^{2}$ | The instantaneous load of EV $k$ upon departing from node $i$ |
| $p_{e}$ | The power of the charging interface |
| $\eta$ | The charging efficiency of the EV |
|  | The duration required to swap an EV battery at the battery swapping station |
| $\sigma_{ik}$ | The charging duration for EV $k$ at charging station $i$ |
| $q_{ik}$ | The charging amount for EV $k$ at charging station $i$ |
| $c_{1}$ | The fixed cost for dispatching an EV |
| $c_{2}$ | EV’s travel cost per unit time |
| $c_{3}$ | The customer service cost per unit time |
| $c_{4}$ | The charging cost per unit time |
| $c_{5}$ | The battery swapping cost for a single replacement |

**Decision variables**

| $x_{ijk}$ | 1 if EV $k$ travels from node $i$ to $j$, 0 otherwise |
| --- | --- |
| $w_{ik}$ | 1 if EV $k$ visits customer $i$, 0 otherwise |
| $y_{ik}$ | 1 if EV $k$ charges at charging station $i$, 0 otherwise |
| $z_{ik}$ | 1 if EV $k$ replaces a battery at battery swapping station $i$, 0 otherwise |
